# Subhepatic Appendicitis: A Systematic Review of Clinical Presentation, Diagnostic Challenges, and Surgical Management

**DOI:** 10.7759/cureus.98002

**Published:** 2025-11-28

**Authors:** Navaneethan Adityaraj Shivalingam Vanaraj, Vishwajit G.V., Keerthika Vijayakumar, Virushnee Senthilkumar, Monish Dharapuram Srinivasan

**Affiliations:** 1 Department of Surgical Oncology, Coimbatore Medical College Hospital, Coimbatore, IND; 2 Department of Community Medicine, Coimbatore Medical College Hospital, Coimbatore, IND; 3 Department of Internal Medicine, Cook County Health, Chicago, USA; 4 Department of Internal Medicine, Coimbatore Medical College Hospital, Coimbatore, IND; 5 Department of General Surgery, Coimbatore Medical College Hospital, Coimbatore, IND

**Keywords:** appendiceal anatomical variants, atypical appendicitis, diagnostic challenge, laparoscopic appendectomy, subhepatic appendicitis, undescended cecum

## Abstract

Subhepatic appendicitis (SHA) is a rare positional variant of appendicitis that frequently mimics hepatobiliary disease, leading to diagnostic confusion and delay. As published evidence is limited to case reports and small series, this systematic review aims to synthesize the clinical presentation, diagnostic pathways, management, and outcomes of reported SHA cases. Following an International Prospective Register of Systematic Reviews (PROSPERO)-registered protocol, PubMed, Scopus, and Google Scholar were searched up to 6 May 2024, with additional eligible reports identified through manual searching and citation chaining. The data extracted were summarized descriptively, and the risk of bias was assessed using the Joanna Briggs Institute tools. Eighty-eight publications (78 case reports, 10 series) describing 97 patients were included. The median age was 29 years, and most patients were adults (71%) and male (69%). Right hypochondriac pain was the predominant symptom (82%), often accompanied by vomiting and fever. Ultrasound identified SHA in roughly one-quarter of cases, whereas CT did so reliably in most cases (81%), improving diagnostic certainty. Nearly all patients underwent surgery, with laparoscopy attempted in about half and occasional conversion to open appendectomy. Intraoperative findings commonly included adhesions, perforation, or abscess. Outcomes were generally favorable, with only a single death reported. A subset of cases demonstrated congenital anomalies such as an undescended cecum, short ascending colon, or malrotation, accounting for the aberrant appendiceal positioning. Clinicians should consider SHA in patients with right hypochondriac pain and inconclusive hepatobiliary investigations. CT improves detection, and timely operative management, often laparoscopic, leads to favorable outcomes.

## Introduction and background

The vermiform appendix is a vestigial organ of the midgut, with multiple possible positions, including retrocecal, subcecal, pelvic, pre-ileal, and post-ileal [1]. Among these, the subhepatic location is rare, where the appendix lies beneath the liver. Congenitally, this abnormal positioning is commonly attributed to midgut malrotation, leading to a subhepatic cecum and appendix. Rarer associations with the subhepatic position, such as renal and gallbladder agenesis, open the possibility of a wider array of pathomechanisms [2]. Subhepatic appendicitis (SHA) was first reported by Jonas in 1921 [3]. Subsequently, cases of SHA have been reported with an annual incidence of 0.09% [4]. This atypical condition has not only led to clinical misdiagnoses but also radiological misdiagnoses, as it frequently mimics hepatobiliary pathology [5].

While some clinicians advocate ultrasonography (USG) as the initial imaging modality due to its availability and non-invasiveness, others prefer computed tomography (CT) for its higher diagnostic accuracy in atypical presentations. However, even CT scans have occasionally failed to detect the inflamed appendix in its subhepatic location, especially when obscured by bowel gas or overlapping hepatic structures. In some cases, the diagnosis has only been confirmed during exploratory laparotomy or laparoscopy, underscoring the condition’s diagnostic ambiguity [6]. Despite multiple case reports, findings are scattered. This is a pioneering systematic review that summarizes and analyzes published cases of SHA, focusing on its clinical presentation, diagnostics, management strategies, and outcomes. By identifying recurring patterns and highlighting diagnostic pitfalls, this review aims to support timely recognition and optimal management of SHA, thereby reducing the risk of avoidable complications.

## Review

Methods

The systematic review was conducted according to the Preferred Reporting Items for Systematic Reviews and Meta-Analyses (PRISMA) guidelines [7] and registered on the International Prospective Register of Systematic Reviews (PROSPERO) (ID: CRD42024543709). Ethical approval was not necessary, as the study is a systematic review.

Information Sources

We conducted a systematic search of PubMed, Scopus, and Google Scholar to identify case reports and small case series describing SHA or an appendix located in a subhepatic position. The primary database search was completed on 6 May 2024, followed by a manual update through 5 October 2025 using citation chaining and Google Scholar alert monitoring.

Search Strategy

Database-specific search strategies without field restrictions were developed to maximize the retrieval of cases of SHA while maintaining specificity:

PubMed: ((subhepatic) OR (sub-hepatic)) AND (((cecum) OR (caecum)) OR (appendix) OR (appendicitis) OR (appendi*))

Scopus: (subhepatic OR "sub-hepatic") AND (cecum OR caecum OR appendix OR appendicitis OR appendi*)

Google Scholar: Manual phrase-based searches combining “subhepatic” and “sub-hepatic” variants across key anatomical terms (appendicitis, appendix, cecum/caecum), complemented by citation chaining and alert-based detection of newly indexed case reports.

Eligibility Criteria

Included studies were case reports or small case series that described an appendix located in a subhepatic position with clinical, radiological, operative, or pathological features consistent with appendicitis. We included cases in which an alternative or concurrent diagnosis (e.g., appendiceal diverticulitis, mucinous neoplasm, B-cell lymphoma) was ultimately identified, as long as the case was clinically reported and managed as SHA, as excluding such cases would under-represent real-world diagnostic presentations.

We excluded: (i) reports without any demographic details (age and sex), as such omissions limit interpretability and may introduce reporting bias; (ii) reports in non-English languages to avoid inaccuracies arising from translated descriptions of pain localization, operative findings, or anatomical variants; (iii) non-original studies; (iv) studies with unavailability of full texts; (v) cadaveric studies; and (vi) cases in which appendiceal inflammation resulted from non-inflammatory mechanical or iatrogenic etiologies (e.g., appendiceal torsion, volvulus of the right colon, post-radiofrequency-ablation reaction) - these cases were narratively described but not quantitatively pooled.

Study Selection

**Figure 1:**
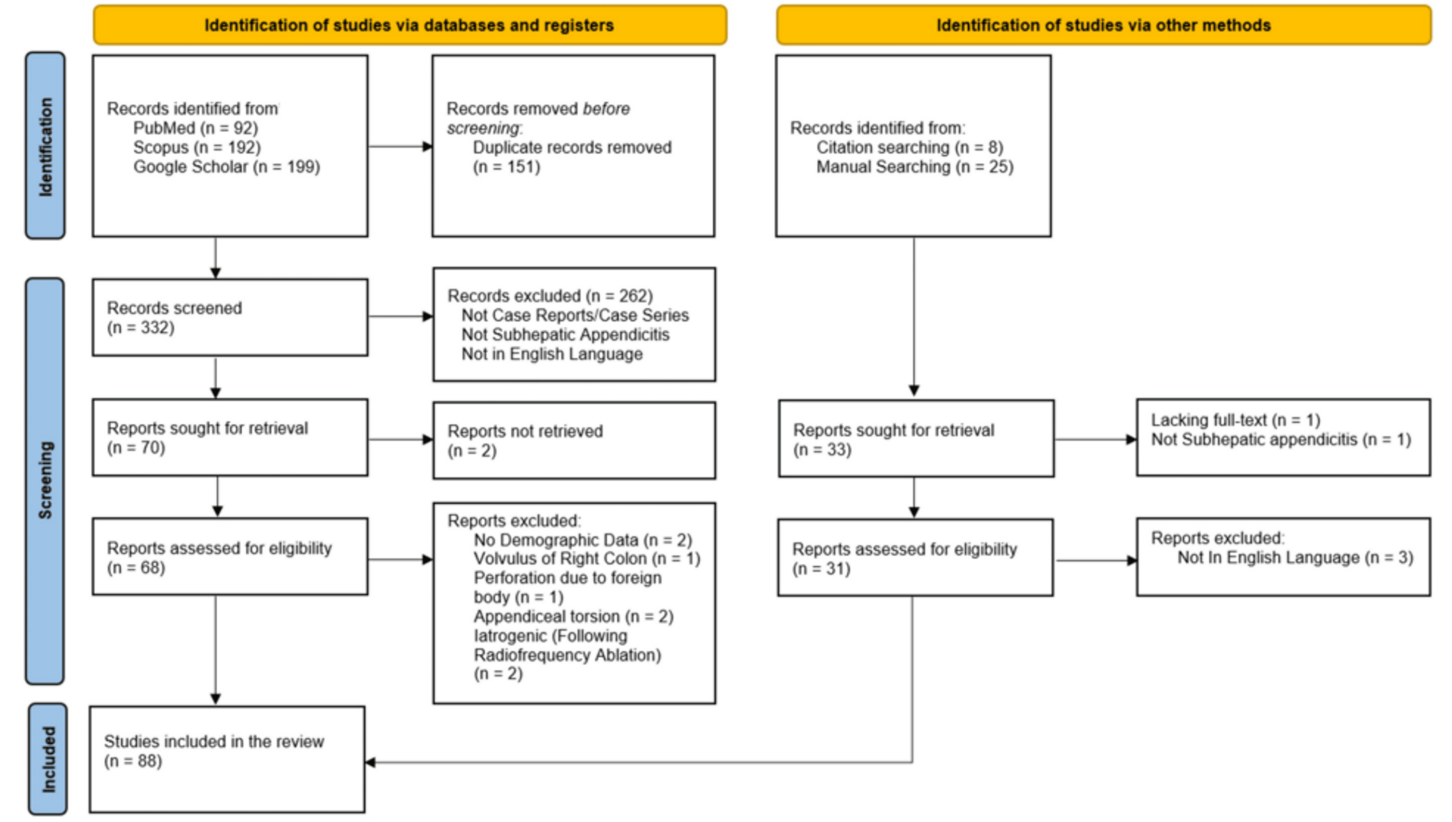
PRISMA 2020 flow diagram depicting the search results from various databases and citation and manual searching PRISMA: Preferred Reporting Items for Systematic Reviews and Meta-Analyses

All search results were imported into Rayyan (a free web-based application) (Rayyan Systems Inc., Cambridge, MA, USA) for duplicate removal and screening [8]. The third, fourth, and fifth authors independently screened titles, abstracts, and full texts against the eligibility criteria. Disagreements were resolved through discussion and adjudication by the first author, serving as an independent third reviewer. A total of 332 non-duplicated records were screened, and 88 studies were included in the pooled analysis, as summarized in the PRISMA 2020 flow diagram (Figure 1).

Data Extraction

Data extraction was conducted independently by the fourth and fifth authors, using a standardized extraction spreadsheet. Extracted variables included demographic characteristics, comorbidities and past medical history, presenting symptoms and complaints, physical examination, laboratory investigations, radiodiagnostic modalities and their findings, confirmatory diagnostic modality, operative approach, intraoperative findings, histopathological results, and postoperative outcomes. Discrepancies were resolved by consensus with the first author. All included case series provided individual patient-level information, allowing every case to be extracted and analyzed separately. No aggregate-only case series were encountered in this review.

Data Handling

Reports containing irreconcilable internal inconsistencies for a variable, such as contradictory demographic or anatomical details, were excluded from analysis of that specific variable as a post-hoc data-cleaning decision not pre-specified in PROSPERO. When a variable was documented in a case report (for example, clinical symptoms or ultrasound findings) but a specific data element within that variable (fever, for example) was not mentioned, the unreported element was interpreted as absent. Conversely, when the variable itself was not described anywhere in the case report, it was coded as “not reported.” Author correspondence was attempted when clarification was required. Missing data were treated strictly as missing, not imputed, and not otherwise inferred. All denominators in the Results, therefore, reflect available-case denominators (n/N) to maintain transparency regarding variable completeness.

Data Synthesis and Analysis

Given the rarity of SHA and the exclusively case-based nature of available evidence, a meta-analysis was neither planned nor feasible. All analyses were purely descriptive. Summary measures included frequencies and percentages, means with standard deviations, medians with interquartile ranges, ranges, and modes, when applicable. SI units were applied consistently throughout.

Assessment of Quality and Risk of Bias (ROB)

The ROB was assessed by the first two authors for all 88 included studies using the Joanna Briggs Institute (JBI) Critical Appraisal Checklist for Case Reports (2020) [9] independently. Each study was evaluated across eight domains addressing reporting clarity on demographics, patient history, clinical presentation, diagnostic findings, treatment interventions, post-treatment condition, adverse events, and key takeaway lessons. To assess the consistency between raters, Cohen’s kappa (κ) was calculated for each domain using the formula:

$\kappa = \frac{P_o - P_e}{1 - P_e}$

Where *P_o_* is the observed agreement, and *P_e_* is the expected agreement due to chance. The strength of agreement for each domain rated was interpreted using Landis and Koch benchmarks (κ < 0 = no agreement; 0.00-0.20 = slight; 0.21-0.40 = fair; 0.41-0.60 = moderate; 0.61-0.80 = substantial; 0.81-1.00 = almost perfect) [10]. The ROB assessment was tabulated for every study included (Table 1).

**Table 1:** Risk of bias (ROB) analysis of the included studies, rated by two different evaluators The risk of bias was assessed for all 88 included studies across eight domains using the Joanna Briggs Institute (JBI) Critical Appraisal Checklist for Case Reports (2020) [9].

Sl. No.	Title	First Author	D1	D2	D3	D4	D5	D6	D7	D8	D1	D2	D3	D4	D5	D6	D7	D8	Reference
			Rater 1	Rater 1	Rater 1	Rater 1	Rater 1	Rater 1	Rater 1	Rater 1	Rater 2	Rater 2	Rater 2	Rater 2	Rater 2	Rater 2	Rater 2	Rater 2	
1	Surgical management of subhepatic perforated appendicitis: a case report	Hakim M	Yes	Yes	Yes	Yes	Yes	Yes	Yes	Yes	Yes	Yes	Yes	Yes	Yes	Yes	Yes	Yes	[1]
2	A rare presentation of acute appendicitis in right upper quadrant caused by renal agenesis	Beh HN	Yes	Yes	Yes	Yes	Yes	Yes	Yes	Yes	Yes	Yes	Yes	Yes	Yes	Yes	Yes	Yes	[2]
3	Undescended cecum and vermiform appendix	Jonas AF	Yes	Yes	Yes	Yes	Yes	Yes	Yes	Yes	Yes	Yes	Yes	Yes	Yes	Yes	Yes	Yes	[3]
4	A case of right upper abdominal pain misdiagnosed on computerized tomography	Singh S	Yes	No	Yes	Yes	Yes	Yes	Yes	Yes	Yes	No	Yes	Yes	Yes	Yes	Yes	Yes	[5]
5	A case of sub hepatic perforated appendicitis presented as multiple gas containing subcapsular hepatic abscesses	Nizamani WM	Yes	Yes	Yes	Yes	Yes	Yes	Yes	Yes	Yes	Yes	Yes	Yes	Yes	Yes	Yes	Yes	[6]
6	A case report: Gangrenous sub-hepatic appendicitis in pregnancy	Abdelkarim S	Yes	Yes	Yes	No	Yes	Yes	Yes	Yes	Yes	Yes	Yes	No	Yes	Yes	Yes	Yes	[11]
7	Perforated subhepatic appendix presenting as gas under diaphragm	Ahangar S	Yes	No	Yes	Yes	Yes	No	No	No	Yes	No	Yes	Yes	Yes	No	Yes	No	[12]
8	Unusual manifestation of acute retrocecal appendicitis: pericholecystic fluid	Algın O	Yes	Yes	Yes	Yes	No	Yes	Yes	Yes	Yes	Yes	Yes	Yes	No	Yes	Yes	Yes	[13]
9	Sub-hepatic appendicitis: case report	Alhoseni M	Yes	Yes	Yes	Yes	Yes	Yes	Yes	Yes	Yes	Yes	Yes	Yes	Yes	Yes	Yes	Yes	[14]
10	Emergency surgical management of sub-hepatic appendicular perforation with abscess; rare presentation of a common disease: a case report	Ali SKS	Yes	Yes	Yes	No	Yes	Yes	Yes	Yes	Yes	Yes	Yes	No	Yes	Yes	Yes	Yes	[15]
11	Subhepatic appendicitis in an 11-year-old boy: a case report	Alqahtani SM	Yes	Yes	Yes	Yes	Yes	Yes	Yes	Yes	Yes	Yes	Yes	Yes	Yes	Yes	Yes	Yes	[16]
12	Malrotated subhepatic caecum with subhepatic appendicitis: diagnostic dilemma: a case report	Anelay BA	Yes	Yes	Yes	Yes	Yes	Yes	Yes	Yes	Yes	Yes	Yes	Yes	Yes	Yes	Yes	Yes	[17]
13	Ultrasonic diagnosis of acute uncomplicated appendicitis located in the subhepatic space	Arbatli MM	Yes	No	Yes	Yes	No	No	No	Yes	Yes	No	Yes	Yes	No	No	No	Yes	[18]
14	Agenesis of the gall bladder, an unexpected finding during laparoscopy; case report	Arif SH	Yes	Yes	Yes	Yes	Yes	Yes	Yes	Yes	Yes	Yes	Yes	Yes	Yes	Yes	Yes	Yes	[19]
15	Hidden appendix: a case report and literature review of perforated acute appendicitis masquerading as acute cholecystitis	Ashwini E	Yes	Yes	Yes	Yes	Yes	Yes	Yes	Yes	Yes	Yes	Yes	Yes	Yes	Yes	Yes	Yes	[20]
16	Subhepatic appendicitis: a diagnostic dilemma	Ball WR	No	Yes	Yes	Yes	Yes	Yes	Yes	Yes	No	Yes	Yes	Yes	Yes	Yes	Yes	Yes	[21]
17	Successful antibiotic treatment of liver abscess in an eight-year-old boy after perforated appendix	Bašković M	Yes	No	Yes	Yes	Yes	Yes	Yes	Yes	Yes	Yes	Yes	Yes	Yes	Yes	Yes	Yes	[22]
18	Duodenal obstruction caused by acute appendicitis with intestinal malrotation in a child	Biçer Ş	Yes	Yes	Yes	Yes	Yes	Yes	Yes	Yes	Yes	Yes	Yes	Yes	Yes	Yes	Yes	Yes	[23]
19	Subhepatic appendicitis	Birchall JD	Yes	No	Yes	Yes	No	No	No	Yes	No	Yes	Yes	No	No	No	No	No	[24]
20	Four pathologies in a single patient with acute abdomen. Who is the culprit? A case report	Chaudhary R	Yes	No	Yes	Yes	Yes	Yes	Yes	Yes	Yes	No	Yes	Yes	Yes	Yes	Yes	Yes	[25]
21	A rare case of perforated “sub-hepatic appendicitis” − a challenging differential diagnosis of acute abdomen based on the combination of appendicitis and maldescent of the caecum	Chiapponi C	Yes	No	Yes	Yes	Yes	Yes	Yes	Yes	Yes	No	Yes	Yes	Yes	Yes	Yes	Yes	[26]
22	Acute appendicitis in the context of undescended cecum: laparoscopic management with restoration of the orthotopic anatomy	Papageorgopoulou C	Yes	No	Yes	No	Yes	Yes	Yes	Yes	Yes	No	Yes	No	Yes	Yes	Yes	Yes	[27]
23	Deep-seated mischief: revealing radiology	Craig JOMC	Yes	No	Yes	Yes	No	No	No	Yes	Yes	No	Yes	Yes	No	No	No	Yes	[28]
24	Appendicitis - is a clinical diagnosis enough?	D’Souza C	Yes	No	Yes	Yes	Yes	Yes	Yes	Yes	Yes	No	Yes	Yes	Yes	Yes	Yes	Yes	[29]
25	Acute perforated appendicitis - an unusual variant simulating acute acalculous cholecystitis	Evans RH	Yes	No	Yes	Yes	Yes	Yes	Yes	Yes	Yes	No	Yes	Yes	Yes	Yes	Yes	Yes	[30]
26	Subhepatic perforated acute appendicitis in a patient with midgut malrotation: a case report and review of the literature	Evola G	Yes	Yes	Yes	Yes	Yes	Yes	Yes	Yes	Yes	Yes	Yes	Yes	Yes	Yes	Yes	Yes	[31]
27	Computed tomography (CT) findings of a diagnostic dilemma: atypically located acute appendicitis	Evrimler S	Yes	No	Yes	Yes	No	No	No	Yes	Yes	No	Yes	Yes	No	No	No	Yes	[32]
28	Uncomplicated subhepatic appendicitis: case report and literature review	Flores-Garcia AE	Yes	No	Yes	Yes	Yes	Yes	Yes	Yes	Yes	No	Yes	Yes	Yes	Yes	Yes	Yes	[33]
29	Subhepatic appendicitis with subdiaphragmatic abscess in a pediatric patient without intestinal malrotation: case report	Galván-Montaño A	Yes	No	Yes	Yes	Yes	Yes	Yes	Yes	Yes	No	Yes	Yes	Yes	Yes	Yes	Yes	[34]
30	Subhepatic appendicitis: a diagnostic conundrum	George BT	Yes	Yes	Yes	Yes	Yes	Yes	Yes	Yes	Yes	Yes	Yes	Yes	Yes	Yes	Yes	Yes	[35]
31	Acute appendicitis in subhepatic location	Giannakopoulou L	Yes	Yes	Yes	Yes	No	Yes	No	Yes	Yes	Yes	Yes	Yes	No	No	No	Yes	[36]
32	An unusual cause of right upper quadrant pain	Hafiz N	Yes	Yes	Yes	Yes	Yes	Yes	Yes	Yes	Yes	No	Yes	Yes	Yes	Yes	Yes	Yes	[37]
33	A cautionary tale of hyperbilirubinaemia in subhepatic appendicitis	Hariharan G	Yes	Yes	Yes	Yes	Yes	Yes	Yes	Yes	Yes	Yes	Yes	Yes	Yes	Yes	Yes	Yes	[38]
34	A very rare case of a subhepatic appendix of 20cm length in a girl	Cherrabi H	Yes	No	No	Yes	No	No	No	No	Yes	No	No	Yes	Yes	No	No	No	[39]
35	Malrotated subhepatic caecum with subhepatic appendicitis: diagnosis and management	Chong HC	Yes	Yes	Yes	Yes	Yes	Yes	Yes	Yes	Yes	Yes	Yes	Yes	Yes	Yes	Yes	Yes	[40]
36	Subhepatic neonatal appendicitis in premature babies: first case detected by ultrasound	Huet F	Yes	Yes	Yes	Yes	No	No	No	Yes	Yes	Yes	Yes	Yes	No	No	No	Yes	[41]
37	Subhepatic appendix: an ectopic topography not to be disregarded: a case report	Ibrahim Mamadou AK	Yes	Yes	Yes	Yes	Yes	Yes	Yes	Yes	Yes	Yes	Yes	Yes	Yes	Yes	Yes	Yes	[42]
38	Perforated sub-hepatic appendix; rare presentation of a common disease	Jaliawala HA	No	Yes	Yes	Yes	Yes	Yes	Yes	Yes	No	Yes	Yes	Yes	Yes	Yes	Yes	Yes	[43]
39	Case report on: appendicitis	Khandar J	Yes	Yes	Yes	Yes	Yes	No	No	Yes	Yes	Yes	Yes	Yes	Yes	No	No	Yes	[44]
40	Subhepatic appendicitis presenting with right upper quadrant pain	Kirresh OZ	Yes	No	No	Yes	No	No	No	Yes	Yes	No	No	Yes	No	No	No	Yes	[45]
41	Laparoscopic management of subhepatic appendicitis	Kılcı BM	Yes	No	Yes	Yes	Yes	Yes	Yes	Yes	Yes	No	Yes	Yes	Yes	Yes	Yes	Yes	[46]
42	Subhepatic perforated subhepatic appendicitis versus acute cholecystitis: a diagnostic dilemma	Koay KL	Yes	Yes	Yes	Yes	Yes	Yes	Yes	Yes	Yes	Yes	Yes	Yes	Yes	Yes	Yes	Yes	[47]
43	Perforated subhepatic appendicitis in the laparoscopic era	Kulvatunyou N	Yes	Yes	Yes	Yes	Yes	Yes	Yes	Yes	Yes	Yes	Yes	Yes	Yes	Yes	Yes	Yes	[48]
44	Subhepatic appendicitis in North-East India: a case series analysis and review of literature	Kumar RK	Yes	No	Yes	Yes	Yes	Yes	Yes	Yes	Yes	No	Yes	Yes	Yes	Yes	Yes	Yes	[49]
45	Classical presentation of acute appendicitis in the case of a subhepatic appendix	Longani SK	Yes	Yes	Yes	Yes	Yes	Yes	Yes	Yes	Yes	Yes	Yes	Yes	Yes	Yes	Yes	Yes	[50]
46	A case report of surgical management of subhepatic acute appendicitis	Pugazhenthi M	Yes	No	Yes	Yes	Yes	Yes	Yes	Yes	Yes	No	Yes	Yes	Yes	Yes	Yes	Yes	[51]
47	Appendicular abscess masquerading as a liver abscess: value of laparoscopy in diagnosis and management	Rangarajan M	Yes	No	Yes	Yes	Yes	Yes	Yes	Yes	Yes	No	Yes	Yes	Yes	Yes	Yes	Yes	[52]
48	Subhepatic acute appendicitis in a 10-year-old male child; typical presentation with atypical location: a case report	Malik S	Yes	No	Yes	Yes	Yes	Yes	Yes	Yes	Yes	No	Yes	Yes	Yes	Yes	Yes	Yes	[53]
49	Acute appendicitis with malrotation of the midgut	Marks JL	Yes	Yes	Yes	Yes	Yes	No	Yes	Yes	Yes	Yes	Yes	Yes	Yes	No	No	No	[54]
50	Subhepatic appendicitis with appendicular diverticulitis: a rare combination for acute abdomen	Mathew M	Yes	Yes	Yes	Yes	Yes	Yes	Yes	Yes	Yes	Yes	Yes	Yes	Yes	Yes	Yes	Yes	[55]
51	A unique case of pediatric subhepatic appendicitis with elevated lipase	McAninch SA	Yes	Yes	Yes	Yes	Yes	Yes	Yes	Yes	Yes	Yes	Yes	Yes	Yes	Yes	Yes	Yes	[56]
52	Encountering a lateral pouch acute appendicitis in pediatric age group: case series study	Almesaibli M	Yes	Yes	Yes	Yes	Yes	Yes	Yes	Yes	Yes	Yes	Yes	Yes	Yes	No	Yes	Yes	[57]
53	High ascending retrocecal appendicitis in a pediatric patient detected by point-of-care ultrasound	Mori T	Yes	Yes	Yes	Yes	Yes	Yes	Yes	Yes	Yes	Yes	Yes	Yes	Yes	Yes	Yes	Yes	[58]
54	Subhepatic appendicitis: a diagnostic dilemma	Kumar N	Yes	No	Yes	Yes	Yes	Yes	Yes	Yes	Yes	Yes	Yes	Yes	Yes	No	Yes	Yes	[59]
55	Subhepatic appendicitis	Oakenful C	Yes	Yes	Yes	Yes	Yes	No	No	Yes	Yes	Yes	Yes	Yes	Yes	No	Yes	Yes	[60]
56	Little rider: giant subhepatic appendicitis	Pawar AP	Yes	Yes	Yes	Yes	Yes	Yes	Yes	Yes	Yes	Yes	Yes	Yes	Yes	Yes	Yes	Yes	[61]
57	Difficult diagnosis of acute abdomen caused by sub hepatic caecum with acute appendicitis - a rare case report	Painuly GP	Yes	Yes	Yes	Yes	Yes	Yes	Yes	Yes	Yes	Yes	Yes	Yes	Yes	Yes	Yes	Yes	[62]
58	Sub hepatic acute appendicitis: a challenging case to diagnose and successfully managed by laparoscopic procedure	Palle P	Yes	No	Yes	Yes	Yes	Yes	Yes	Yes	Yes	No	Yes	Yes	Yes	Yes	Yes	Yes	[63]
59	Subhepatic appendix with fecalith mimicking acute cholecystitis with gallstone	Patel NR	Yes	Yes	Yes	Yes	Yes	Yes	Yes	Yes	Yes	No	Yes	Yes	Yes	No	Yes	Yes	[64]
60	Unusual pneumonia mimic	Pires JR	Yes	Yes	Yes	Yes	Yes	Yes	Yes	Yes	Yes	Yes	Yes	Yes	No	Yes	Yes	Yes	[65]
61	Subhepatic appendicitis: diagnostic dilemma: a case report	Singh R	Yes	No	Yes	Yes	Yes	Yes	Yes	Yes	Yes	Yes	Yes	Yes	Yes	No	Yes	Yes	[66]
62	Subhepatic appendicitis	Rappaport WD	Yes	No	Yes	Yes	Yes	Yes	Yes	Yes	Yes	No	Yes	Yes	Yes	Yes	Yes	Yes	[67]
63	Subhepatic appendicitis masquerading as acute cholecystitis: a lesson learnt!	Rodrigues G	Yes	Yes	Yes	Yes	Yes	No	Yes	No	Yes	Yes	Yes	Yes	Yes	Yes	Yes	No	[68]
64	Rare appendiceal escapades in childhood: the Grande experience!	Shrestha AL	Yes	No	Yes	Yes	Yes	Yes	Yes	Yes	Yes	No	Yes	Yes	Yes	Yes	Yes	Yes	[69]
65	Appendix and diagnostic mystery: subhepatic appendicitis	Soomro N	Yes	Yes	Yes	Yes	Yes	Yes	Yes	Yes	Yes	Yes	Yes	Yes	Yes	Yes	Yes	Yes	[70]
66	An unexpected discovery	Stancu S	Yes	Yes	Yes	Yes	Yes	Yes	Yes	Yes	Yes	Yes	Yes	Yes	Yes	Yes	Yes	Yes	[71]
67	Acute appendicitis in a patient with sub-hepatic, sub-serosal, and retroperitoneal location. An intraoperative management challenge	Teferi DA	Yes	Yes	Yes	Yes	Yes	Yes	Yes	Yes	Yes	Yes	Yes	Yes	Yes	Yes	Yes	Yes	[72]
68	Subhepatically located appendicitis due to adhesions: a case report	Ting JYS	Yes	Yes	Yes	Yes	Yes	Yes	Yes	Yes	Yes	Yes	Yes	Yes	Yes	Yes	Yes	Yes	[73]
69	Sub diaphragmatic abscess leading to empyema thoracis in a case of perforated appendix: a rare case report	Rajgopal T	Yes	No	Yes	Yes	Yes	Yes	Yes	Yes	Yes	No	Yes	Yes	Yes	Yes	Yes	Yes	[74]
70	The diagnosis of atypically situated acute appendicitis	Tseng H-C	Yes	Yes	Yes	Yes	Yes	Yes	Yes	Yes	Yes	Yes	Yes	Yes	Yes	No	No	Yes	[75]
71	Subhepatic appendicitis a rare entity	Vadher DP	Yes	Yes	Yes	Yes	Yes	Yes	Yes	Yes	Yes	Yes	Yes	Yes	Yes	Yes	Yes	Yes	[76]
72	Perforated subhepatic appendix presenting as a gas under diaphragm: clinical diagnostic challenges for surgical residents: a case report	Yadav DK	Yes	Yes	Yes	Yes	Yes	Yes	Yes	Yes	Yes	Yes	Yes	Yes	Yes	Yes	Yes	Yes	[77]
73	Subhepatic appendicitis presenting with recurrent abdominal pain	Yousef AH	Yes	Yes	Yes	Yes	Yes	Yes	Yes	Yes	Yes	Yes	Yes	Yes	Yes	Yes	Yes	Yes	[78]
74	Perforated subhepatic appendicitis: a case report	Muhamad Zin MH	Yes	No	Yes	Yes	Yes	Yes	Yes	Yes	Yes	Yes	Yes	Yes	Yes	Yes	Yes	Yes	[79]
75	Subhepatic appendicitis in a 27-year-old male: a case report from Odahulle General Hospital of Ethiopia	Abagojam AHA	Yes	Yes	Yes	Yes	Yes	No	Yes	Yes	Yes	Yes	Yes	Yes	Yes	Yes	Yes	Yes	[80]
76	Case report: subhepatic appendicitis complicated by hepatic abscess as the initial presentation of primary diffuse large B-cell lymphoma of the appendix: first reported case	Chen H	Yes	Yes	Yes	Yes	Yes	Yes	Yes	Yes	Yes	Yes	Yes	Yes	Yes	Yes	No	Yes	[81]
77	Synchronous acute appendicitis and cholecystitis	Aljunaydil AA	Yes	Yes	Yes	Yes	Yes	Yes	Yes	Yes	Yes	Yes	Yes	Yes	Yes	Yes	Yes	Yes	[82]
78	Open appendectomy for subhepatic appendicitis in an adult male: a case report	Chaudhary R	No	Yes	No	Yes	Yes	Yes	Yes	Yes	No	Yes	No	Yes	Yes	Yes	Yes	Yes	[83]
79	Diagnostic dilemma in pediatric subhepatic appendicitis: a report of two cases	Nusrath MP	Yes	Yes	Yes	Yes	Yes	Yes	Yes	Yes	Yes	Yes	Yes	Yes	Yes	Yes	Yes	Yes	[84]
80	A rare case of subhepatic acute appendicitis: a case report	Tesemma A	Yes	Yes	Yes	Yes	Yes	Yes	Yes	Yes	Yes	Yes	No	No	Yes	Yes	Yes	Yes	[85]
81	Synchronous appendicitis and cholecystitis in the setting of intestinal malrotation: a rare presentation of adhesion of the appendix to the gallbladder	Lee K	Yes	Yes	Yes	Yes	Yes	Yes	Yes	Yes	Yes	Yes	Yes	Yes	Yes	Yes	Yes	Yes	[86]
82	Peculiarities of diagnosis and management for subhepatic appendicitis	Soh GT	Yes	No	Yes	No	Yes	Yes	Yes	Yes	Yes	Yes	Yes	No	Yes	Yes	Yes	Yes	[87]
83	Incomplete common mesentery revealed by appendicitis in adults: a case report	Oubihi A	No	Yes	No	Yes	Yes	Yes	Yes	Yes	No	Yes	No	Yes	Yes	Yes	Yes	Yes	[88]
84	Concurrent acute appendicitis and epiploic appendagitis: a rare clinical case	Al Khalili H	No	Yes	Yes	No	No	Yes	Yes	Yes	No	Yes	Yes	No	No	No	Yes	Yes	[89]
85	Subhepatic perforated appendicitis complicated by intestinal obstruction: a case report	Ullah W	Yes	Yes	Yes	Yes	Yes	Yes	Yes	Yes	Yes	Yes	Yes	Yes	Yes	Yes	Yes	Yes	[90]
86	Well differentiated appendiceal neuroendocrine neoplasm an incidental finding post subhepatic appendectomy: a case report and review of the literature	Allafi FA	Yes	Yes	Yes	Yes	Yes	Yes	Yes	Yes	Yes	Yes	Yes	Yes	Yes	Yes	Yes	Yes	[91]
87	A retrocaecal appendix presenting with recurrent right upper quadrant pain (RUQ): a rare presentation of a rather common surgical pathology	Ferrie A	Yes	Yes	Yes	Yes	Yes	Yes	Yes	Yes	Yes	Yes	Yes	No	Yes	Yes	Yes	Yes	[92]
88	A rare case of long subserosal paracolic subhepatic appendix	Ranga H	Yes	Yes	Yes	Yes	Yes	Yes	Yes	Yes	Yes	Yes	Yes	Yes	Yes	Yes	Yes	Yes	[93]

**Figure 2:**
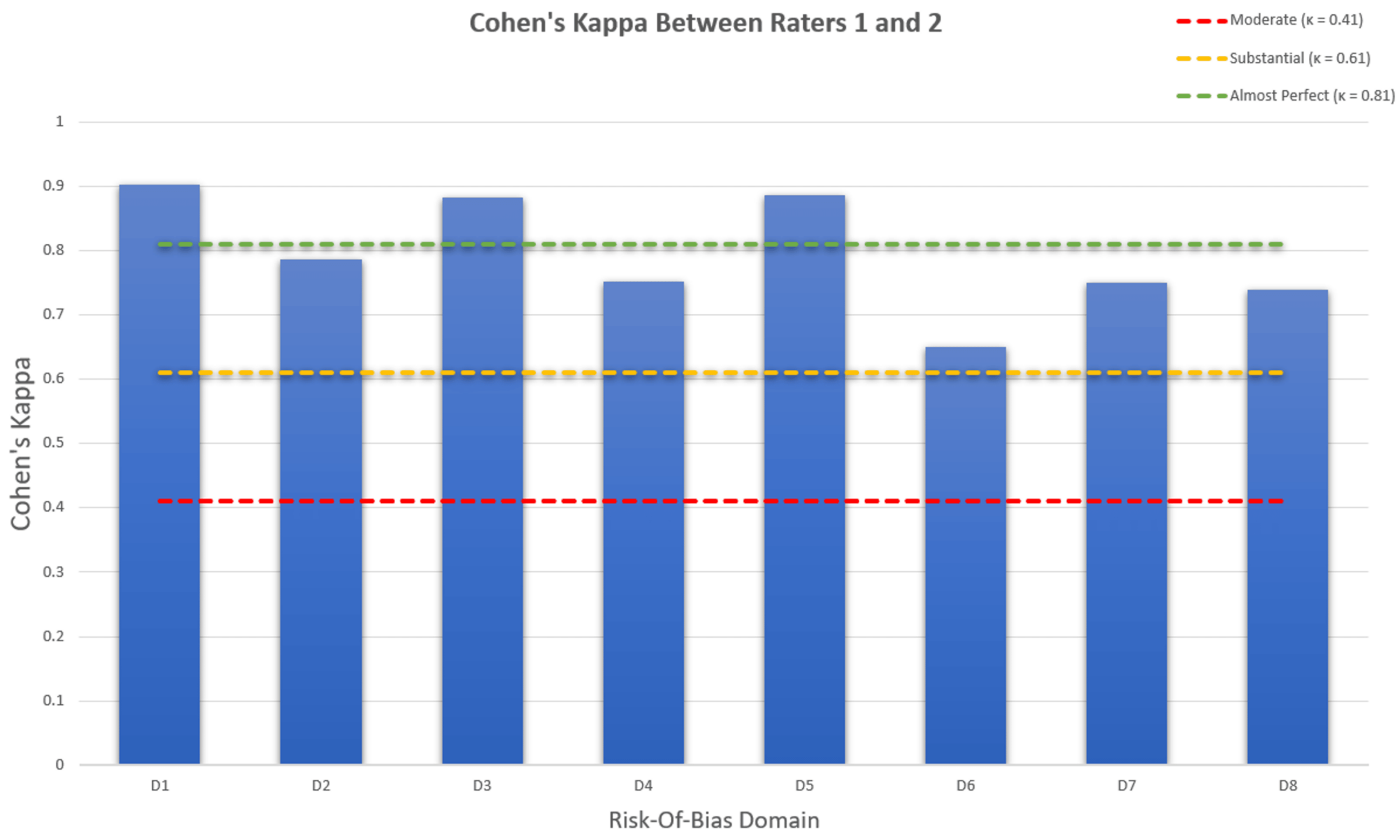
Cohen’s kappa agreement (κ) between two independent raters across eight domains of the risk of bias assessment The strength of agreement, κ, was interpreted using Landis and Koch benchmarks (κ = 0.41-0.60 = moderate; 0.61-0.80 = substantial; 0.81-1.00 = almost perfect) [10].

The resulting κ values indicated almost perfect agreement for D1, D3 and D5 (κ = 0.883-0.903) and substantial agreement for D2, D4, and D6-D8 (κ = 0.650-0.787) (Figure 2). Although the JBI Critical Appraisal Checklist for Case Reports does not provide a formal scoring system, items were assigned 1 point for "Yes" and 0 for "No." Based on cumulative scores (maximum of 16 for two raters across eight domains), studies were categorized as low risk (score ≥ 13), moderate risk (score 9-12), or high risk (score ≤ 8). These tallied scores were used only descriptively to summarize reporting completeness and should not be interpreted as validated quality grades. As such, the distribution showed 72 studies (81.8%) with a low risk, 10 (11.4%) with a moderate risk, and six (6.8%) with a high ROB.

Results

Eighty-eight studies (78 case reports and 10 case series) were included in the final analysis. A total of 97 patients with SHA were identified. The key findings are elaborated and presented in Table 2. The complete pooled dataset, including all summary measures described in the Results section, is presented in Appendices A-B.

**Table 2:** Core results of the systematic review SGPT: serum glutamic-pyruvic transaminase; ALT: alanine aminotransferase

Variable	Number (%) of patients
Sex (n = 97)	
Male	67 (69.07)
Female	30 (30.93)
Age (n = 93)	Years
Range	0.0055 (2 days) to 71
Mean	30.56 (SD = 18.02)
Age Category (n = 96)	
Adults	68 (70.83)
Pediatric	24 (29.17)
Symptoms (n = 96)	
Pain	93 (96.88)
Site of Pain (n = 93)	
Abdomen	91 (97.85)
Right Flank	8 (8.60)
Site of Abdominal Pain (n = 82)	
Right Hypochondrium	67 (81.71)
Right Iliac Fossa	44 (53.36)
Nature of Pain (n = 52)	
Migratory	16 (30.77)
Periumbilical/Umbilical to Right Upper Quadrant	10 (62.50)
Periumbilical/Umbilical to Right Lower Quadrant	9 (56.25)
Radiating	10 (19.23)
To the back	3 (30.00)
To the Right Flank	3 (30.00)
To the Right Iliac Fossa	3 (30.00)
Continuous/Persistent/Constant	9 (17.31)
Colicky	8 (15.38)
Recurrent	6 (11.54)
Vomiting/Regurgitation	66 (68.75)
Fever	45 (46.88)
Nausea	32 (33.33)
Loss of Appetite	19 (19.79)
Clinical Signs (n = 92)	
Febrile	36 (38.30)
Tenderness (n = 84)	84 (91.30)
Right Hypochondrium	67 (79.76)
Right Iliac Fossa	38 (45.24)
Rebound Tenderness (n = 26)	26 (28.26)
Right Iliac Fossa	17 (65.38)
Right Hypochondrium	16 (61.54)
Guarding (n = 30)	30 (32.61)
Right Hypochondrium	21 (70.00)
Right Iliac Fossa	19 (63.33)
Laboratory Investigations (n = 88)	
Reporting of Leucocytosis (n = 84)	Present in 64 (76.19)
Reporting of Neutrophilia (n = 87)	Present in 38 (43.68)
Reporting of CRP (n = 21, 23.86%)	
Range (mg/L)	12.4-347
Mean (mg/L)	147 (SD = 117.2)
Hyperbilirubinemia	7 (7.95)
Conjugated Hyperbilirubinemia	3 (3.41)
Elevated SGPT/ALT	3 (3.41)
Ultrasound Abdomen (n = 78)	
Abnormal Study	61 (78.21)
Appendix located Subhepatically	21 (26.92)
Computed Tomography (CT) (n = 52)	
Type of Study (n = 52)	
Contrast-Enhanced	33 (71.15)
Plain	10 (19.23)
Not Reported	5 (9.62)
Subhepatic Appendix Reported	42 (80.77)
Appendix Thickened/Dilated	22 (42.31)
Periappendiceal Fat Stranding	19 (36.54)
Subhepatic/Undescended Caecum	15 (28.85)
Appendicolith	15 (28.85)
Appendix Perforated	11 (21.15)
Collection in Right Subhepatic Space	10 (19.23)
Retrocecal Appendix	11 (21.15)
Presence/Suspicion of Malrotation	4 (7.69)
Preoperative Diagnoses (n = 89)	
Appendicitis	69 (77.53)
Subhepatic Appendicitis	58 (65.17)
Cholecystitis	25 (28.09)
Confirmatory Diagnostic Method (n = 97)	
Computed Tomography (CT)	39 (40.21)
Ultrasound Abdomen	18 (18.56)
Intraoperatively	38 (39.18)
Laparoscopically	13 (34.21), (13.40)
Via Laparotomy	25 (65.79), (25.77)
Management (n = 97)	
Medical/Conservative	3 (3.09)
Surgical	93 (95.88)
Type of Appendectomy Reported (n = 85)	(91.40)
Laparoscopic Appendectomy Attempted	44 (51.76)
Laparoscopic Appendectomy Converted to Open Appendectomy	7 (15.91)
Open Appendectomy	48 (56.47)
Peritoneal Toileting/Lavage	23 (27.06)
Placement of Surgical Drain	24 (28.24)
Intraoperative Findings (n = 85)	
Presence of Adhesions	33 (38.82)
Perforated Appendix	26 (30.59)
Presence of Abscess/Pus (n = 26)	26 (30.59)
Subhepatic Space/Morison’s Pouch	14 (53.85)
Retrocecal Appendix	20 (23.53)
Gangrenous Appendix	12 (14.12)
Presence of Appendicolith	10 (11.76)
Bacterial Culture of Pus (n = 8)	
Organism Isolated: *Escherichia coli*	All 8 cases
Postoperative Complications (n = 83)	
None	72 (86.75)
Surgical Site Infection	3 (3.61)
Histopathology Report (n = 41)	
Acute Appendicitis	28 (68.29)
Outcomes (n = 85)	
Discharged	84 (98.82)
Died	1 (1.18)
Postoperative Day of Discharge for Laparoscopic Appendectomy (n = 20)	
Range	1st-9th day
Mean	3.1 days (SD = 2.25)
Postoperative Day of Discharge for Open Appendectomy (n = 35)	
Range	1st-32nd day
Mean	6.11 days (SD = 6.08)

Patient Characteristics

The pooled cohort was young, with a mean and median age of 30.56 and 29 years, respectively (range = 2 days to 71 years; n = 93). Adults accounted for 70.83% (68/96), while pediatric patients comprised 29.17% (24/96). There was a male predominance (67/97, 69.07%), and female patients comprised 30.93% (30/97).

Clinical Presentation

Pain was nearly universal (93/96, 96.88%). Abdominal pain was noted in the vast majority (91/93, 97.85%). It was predominantly present in the right hypochondrium (67/82, 81.71%), followed by the right iliac fossa (44/82, 53.36%) when the site was specified. Among the 52 reports that detailed its nature, pain was migratory in 16 (30.77%), most often from the periumbilical or umbilical region to the right upper or lower quadrant; radiating in 10 (19.23%), typically to the back, right flank, or iliac fossa; and continuous or persistent in nine (17.31%). Colicky pain was reported in eight (15.38%) and recurrent pain in six (11.54%). Associated symptoms included vomiting (66/96, 68.75%), fever (45/96, 46.88%), nausea (32/96, 33.33%), and loss of appetite (19/96, 19.79%). On examination, 36/92 (38.30%) were febrile. Abdominal tenderness was the predominant sign (84/92, 91.30%), most often elicited in the right hypochondrium (67/84, 79.76%). Guarding occurred in 30/92 (32.61%) and rebound tenderness in 26/92 (28.26%), both mainly in the right hypochondrium or iliac fossa.

Laboratory Investigations

Leukocytosis was present in 64/84 (76.19%), and neutrophilia in 38/87 (43.68%). C-reactive protein (CRP) was reported in 21 cases (23.86%), with a mean value of 147 mg/L (range 12.4-347 mg/L). Hyperbilirubinemia occurred in 7/88 (7.95%), and conjugated hyperbilirubinemia in three (3.41%). Transaminitis (elevated serum glutamic-pyruvic transaminase (SGPT), also known as alanine aminotransferase (ALT)) was also reported in three cases. Although the laboratory profile broadly reflected an inflammatory process, the markers were nonspecific and varied widely, limiting their value in distinguishing SHA from other causes of right upper quadrant or hypochondriac pain.

Imaging and Preoperative Diagnosis

USG was performed in 78 patients, identifying an abnormal study in 61 (78.21%), but a subhepatic appendix in only 21 (26.92%). CT was performed in 52 cases and was the most reliable imaging modality, confirming a subhepatic appendix in 42 (80.77%). Common CT features included appendiceal thickening or dilatation (22/52, 42.31%), periappendiceal fat stranding (19/52, 36.54%), undescended cecum (15/52, 28.85%), and appendicolith (15/52, 28.85%). Preoperative misdiagnosis was frequent: cholecystitis was suspected in 25/89 (28.09%), and other hepatobiliary or upper gastrointestinal conditions in several additional cases. The review identifies CT to be superior to USG in establishing the diagnosis, and comparative identification was seen intraoperatively as well, highlighting the persistent diagnostic challenges.

Management

Almost all patients underwent surgery (93/97, 95.88%), with only three managed conservatively. Of the 85 cases specifying surgical technique, laparoscopic appendectomy was attempted in 44 (51.76%), with conversion to open surgery in seven (15.91%), mainly due to dense adhesions or difficulty localizing the appendix in the subhepatic position. Open appendectomy was performed in 48 (56.47%). Peritoneal lavage and drains were used in 23 (27.06%) and 24 (28.24%) cases, respectively.

Intraoperative Findings

Adhesions were common, 33/85 (38.82%), and so were complications of delayed presentation - perforation and pus/abscess collections were both documented in 26/85 (30.59%). Gangrenous change and appendicolith were noted in a minority (12/85 (14.12%) and 10/85 (11.76%), respectively. Microbiology from pus, when cultured (n = 8), yielded *Escherichia coli* in all cases.

Outcomes

The postoperative course was favorable in nearly all patients. A total of 84/85 (98.82%) were discharged after uneventful recovery. Postoperative complications were uncommon, the most frequent being surgical site infection (3/83, 3.61%), present in a minute proportion. There was one death (1.18%) following refusal to undergo operative management. Autopsy confirmed the presence of a subhepatic cecum and a gangrenous appendix, as well as the presence of extensive adhesions and an abscess in the right hypochondrium. Where reported, the length of stay averaged 3.1 days after laparoscopic and 6.11 days after open appendectomy, indicating a shorter recovery with the minimally invasive approach.

Histopathology

Histopathology was reported in 41 cases, confirming acute appendicitis in 28 (68.29%). Isolated reports mentioned a case each of diffuse large B-cell lymphoma and a well-differentiated appendiceal neuroendocrine neoplasm, both found incidentally.

Congenital Anatomic Associations

A subset of 51 cases explicitly documented congenital anomalies such as an undescended or subhepatic cecum, intestinal malrotation, or short ascending colon (Table 3).

**Table 3:** Core results of the cases of subhepatic appendicitis with documented congenital anatomical anomalies CRP: C-reactive protein; SGPT: serum glutamic-pyruvic transaminase; ALT: alanine aminotransferase

Variable	Number (%) of patients
Sex (n = 51)	
Male	38 (74.51)
Female	13 (25.49)
Age (n = 50)	Years
Range	7-71
Mean	33.28 (SD = 16.48)
Age Category (n = 51)	
Adults	42 (82.35)
Paediatric	9 (17.65)
Symptoms (n = 51)	
Pain	50 (98.04)
Site of Pain (n = 50)	
Abdomen	50 (100.00)
Right Flank	5 (10.00)
Site of Abdominal Pain (n = 46)	
Right Hypochondrium	35 (76.09)
Right Iliac Fossa	23 (50.00)
Nature of Pain (n = 28)	
Migratory	9 (32.14)
Periumbilical/Umbilical to Right Upper Quadrant	6 (66.67)
Periumbilical/Umbilical to Right Lower Quadrant	5 (55.56)
Periumbilical to Right Flank	1 (11.11)
Radiating	6 (20.69)
To the Back or to the Right Flank or to the Right Iliac Fossa	2 (33.33) Each
To the Lower Abdomen	1 (16.67)
Continuous/Persistent/Constant	6 (21.43)
Progressive in Intensity	4 (14.29)
Recurrent	3 (10.71)
Vomiting/Regurgitation	33 (64.71)
Fever	19 (37.25)
Nausea	21 (41.18)
Loss of Appetite	12 (23.53)
Clinical Signs (n = 49)	
Febrile	15 (29.41)
Tenderness (n = 46)	46 (93.88)
Right Hypochondrium	40 (86.96)
Right Iliac Fossa or Right Lumbar	17 (36.96)
Rebound Tenderness (n = 16)	16 (32.65)
Right Hypochondrium	10 (62.50)
Right Iliac Fossa	9 (56.25)
Guarding (n = 16)	16 (32.65)
Right Hypochondrium	11 (68.75)
Right Iliac Fossa	8 (53.33)
Laboratory Investigations (n = 45)	
Reporting of Leucocytosis (n = 45)	Present in 36 (80.00)
Reporting of Neutrophilia (n = 45)	Present in 19 (42.22)
Reporting of CRP (n = 7, 15.56%)	
Range (mg/L)	28 – 347
Mean (mg/L)	162 (S.D = 119.1)
Hyperbilirubinemia	3 (6.67)
Conjugated Hyperbilirubinemia	1 (2.22)
Elevated SGPT/ALT	1 (2.22)
Ultrasound Abdomen (n = 38)	
Abnormal Study	28 (73.68)
Appendix located Subhepatically	7 (18.42)
Collection/Free Fluid in Right Subhepatic Space/Morison's Pouch	7 (18.42)
Computed Tomography (CT) (n = 30)	
Type of Study (n = 30)	
Contrast-Enhanced	24 (80.00)
Plain	3 (10.00)
Not Reported	3 (10.00)
Subhepatic Appendix Reported	25 (83.33)
Subhepatic/Undescended Caecum	15 (50.00)
Appendix Thickened/Dilated	11 (36.67)
Periappendiceal Fat Stranding	10 (33.33)
Appendix Perforated	8 (26.67)
Collection in Right Subhepatic Space	7 (23.33)
Appendicolith	7 (23.33)
Retrocecal Appendix	6 (20.00)
Presence/Suspicion of Malrotation	4 (13.33)
Preoperative Diagnoses (n = 45)	
Appendicitis	36 (80.00)
Subhepatic Appendicitis	31 (68.89)
Cholecystitis	17 (37.78)
Confirmatory Diagnostic Method (n = 51)	
Computed Tomography (CT)	25 (49.02)
Ultrasound Abdomen	6 (11.76)
Intraoperatively	19 (37.25)
Laparoscopically	6 (31.58), (11.76)
Via Laparotomy	13 (68.42), (25.49)
Management (n = 51)	
Medical/Conservative	1 (1.96)
Surgical	49 (96.08)
Type of Appendectomy Reported (n = 49)	
Laparoscopic Appendectomy Attempted	27 (55.10)
Laparoscopic Appendectomy Converted to Open Appendectomy	3 (11.11)
Open Appendectomy	25 (51.02)
Peritoneal Toileting/Lavage	14 (28.57)
Placement of Surgical Drain	14 (28.57)
Intraoperative Findings (n = 47)	
Presence of Adhesions	20 (42.55)
Perforated Appendix	15 (31.91)
Presence of Abscess/Pus (n = 13)	13 (27.66)
Subhepatic Space/Morison’s Pouch	8 (61.54)
Retrocecal Appendix	11 (23.40)
Gangrenous Appendix	6 (12.77)
Short Ascending Colon	7 (14.89)
Presence of Appendicolith	4 (8.51)
Bacterial Culture of Pus (n = 1)	
Organism Isolated: *Escherichia coli*	1 case
Postoperative Complications (n = 49)	
None	40 (81.63)
Surgical Site Infection	2 (4.08)
Histopathology Report (n = 23)	
Acute Appendicitis	17 (73.91)
Outcomes (n = 48)	
Discharged	47 (97.92)
Died	1 (2.08)
Postoperative Day of Discharge for Laparoscopic Appendectomy (n = 20)	
Range	1st-7th day
Mean	3.14 days (SD = 1.95)
Postoperative Day of Discharge for Open Appendectomy (n = 35)	
Range	1st-20th day
Mean	5.94 days (SD = 5.17)

These patients were marginally older on average (mean 33.28 years, range 7-71) and predominantly male (38/51, 74.51%). Adults comprised 82.35% of this subgroup.

Pain remained almost universal (50/51, 98.04%) and was localized mainly to the right hypochondrium (35/46, 76.09%) or right iliac fossa (23/46, 50.00%). Associated symptoms such as fever (37.25%) and vomiting (64.71%) were less common than in the overall cohort, while loss of appetite (23.53%) and nausea (41.18%) were greater in proportion. Tenderness was present in 46/49 (93.88%), with guarding and rebound tenderness in approximately one-third. All three signs were predominantly elicited in the right hypochondrium.

Laboratory abnormalities were comparable: leukocytosis in 36/45 (80.00%), neutrophilia in 19/45 (42.22%), and CRP elevation in seven (15.56%; mean 162 mg/L). Hyperbilirubinemia was reported in three (6.67%).

USG was limited (subhepatic appendix visualized in 7/38, 18.42%), while CT imaging revealed characteristic congenital findings - undescended cecum in 15/30 (50.00%), malrotation in four (13.33%), and subhepatic appendix in 25/30 (83.33%). Cholecystitis was again a common preoperative misdiagnosis (17/45, 37.78%).

Surgery was the mainstay (49/51, 96.08%). Laparoscopic appendectomy was attempted in 27 (55.10%), with conversion in three (11.11%). Open appendectomy was performed in 25 (51.02%), often with lavage or drain placement (28.57% each). Intraoperatively, adhesions (42.55%) were the most common finding, followed by perforation (31.91%) and abscess (27.66%). A short ascending colon was described in seven (14.89%) - reinforcing the congenital basis for the subhepatic appendix. These findings underscore that congenital variants of SHA share similar inflammatory complications with the overall cohort, while additionally demonstrating anatomic features consistent with aberrant cecal location.

Outcomes were similarly favorable: 81.63% had an uneventful postoperative course, and 47/48 (97.92%) were discharged. Mortality was 2.08% (n = 1). Mean postoperative stay was 3.14 days in laparoscopic and 5.94 days in open appendectomies, respectively.

Taken together, this subgroup highlights cases in which the congenital basis of SHA was explicitly demonstrated. Their clinical presentation was broadly similar to the overall cohort, but imaging and operative findings yielded clearer embryological anomalies. These data reinforce the congenital underpinnings of SHA while also underscoring persistent diagnostic overlap with hepatobiliary disease.

Discussion

The present systematic review analyzed 88 studies, encompassing a total of 97 cases of SHA. Right hypochondriac pain, leukocytosis, and non-diagnostic hepatobiliary USG were common clues towards SHA. CT scan was the most commonly used confirmatory diagnostic modality. Surgical management was the mainstay of treatment, with laparoscopic appendectomy attempted in just over half of the cases. Conversions to open surgery were noted due to complications such as dense adhesions. Patients undergoing open procedures demonstrated a modestly longer postoperative stay, reflecting the technical challenges associated with aberrant cecal and appendiceal positioning. Postoperative outcomes were largely favorable, as most patients were discharged with a low postoperative complication rate, although one patient succumbed to their condition. A subgroup of 51 cases in the review explicitly reported congenital anatomical anomalies, reinforcing the embryological basis of SHA. Cases of congenital SHA were more frequently misdiagnosed as cholecystitis. Imaging, particularly CT, often revealed an undescended cecum, while intraoperative findings occasionally documented a short ascending colon. Clinical presentation and complication rates in the subgroup closely resembled those of the overall cohort.

Etiology

During midgut elongation, a hairpin-shaped loop is formed with the superior mesenteric artery dividing it into the pre-arterial and post-arterial segments. Being unable to accommodate the entire structure, a physiological hernia occurs. The primordial structure of the appendix and cecum appears as a conic bud in the distal or post-arterial segment. This conic bud acts as the border between the ileum and colon. A total of 270-degree counterclockwise midgut rotation occurs, divided into phases of 90-degree and 180-degree, along with its involution into the body. As a result, the conic bud comes to lie on the right side under the liver, and the elongation of the transverse colon pushes it against the body wall. The conic bud is then pushed caudally due to proximal colonic elongation. Hence, the cecum descends into the right iliac fossa [94]. Therefore, the final appendiceal location is determined by midgut rotation and movement, which, if disturbed, can lead to aberrant positioning. Subhepatic appendix has also been associated with right kidney and gallbladder agenesis [2,19].

Iatrogenic SHA has been reported following radiofrequency ablation (RFA) of tumors located in the inferior segments of the liver, with subsequent abscess and fistula formation between the RFA segment and the appendix tip. The proximity of the subhepatic appendix to the RFA site and its thin wall predispose it to collateral thermal damage [95]. Mechanical causes of appendiceal inflammation have also been documented, including a fatal case of volvulus of the right colon with a subhepatic cecum leading to secondary inflammation and strangulation of the appendix and death due to postoperative shock following a mesenteric infarct [96], and two reports describing torsion of the subhepatic appendix resulting in its necrosis [97,98], all of which may highlight vascular compromise from torsion or volvulus as a distinct mechanical pathogenesis in contrast to the typical luminal obstruction-based inflammatory process.

Diagnostic Considerations

This review depicts cases involving diverse patient demographics, with the majority presenting with abdominal pain localized to the right hypochondrium, thereby commonly mimicking hepatobiliary disease.

Rarely, symptomatology and signs exhibited due to the aberrant location of the appendix can even mimic other gastric, intestinal, and even pulmonary diseases [20,99]. A case presenting with pleuritic chest pain along with right upper quadrant pain and chest X-ray showing right lower pulmonary lobe infiltrate was misdiagnosed and treated as pneumonia. The patient progressed to peritonitis, prompting an exploratory laparoscopy, and only then was the SHA caught. This was explained by the passage of bacteria to the thorax due to the pressure gradient and lymphatic flow, prompting a pulmonary inflammatory response [65].

Interestingly, Murphy’s sign was elicited in a case of SHA in a patient with a prior history of cholecystectomy for suspected biliary colic, and has again presented with persistent symptoms [37]. Hence, we cannot preclude the possibility of a presentation with right iliac fossa tenderness, where an expected classical positioning has been met by an intra-operative surprise of a subhepatic appendix [37,49]. However, most cases that presented with right hypochondriac pain were consistent with the underlying location.

In some instances of subhepatic appendix, hyperbilirubinemia and elevated lipase have been wrongly attributed to gallbladder pathologies and pancreatitis, respectively [20,56]. Physical findings and laboratory investigations may be misleading in atypical cases, giving other modalities such as radiology and diagnostic laparoscopy an important role.

USG has long been recognized as a first-line imaging modality in the diagnosis of acute appendicitis. However, it has reduced sensitivity compared to CT, particularly in the adult population [100]. The latest guidelines recommend a combination of comprehensive clinical indicators and ultrasound to reduce the need for CT [100,101]. Factors such as cost and radiation exposure have influenced this protocol; hence, the use of low-dose CT is being explored as a first-line measure [102]. The value of a negative USG has to be questioned due to its operator-dependent nature and the possibility of aberrant pathologies [100]. On USG, the vermiform structure showed a display rate of 90% in the standard position, whereas only 27.8% in the case of SHA, forcing us to rely on other ultrasonic indicators, such as the fishbone sign, which can be an important diagnostic clue, whose presence we cannot take for granted [101].

Even with the limitations of USG, authors have highlighted the utility of initial point-of-care ultrasonography (POCUS) in the diagnosis of atypically located appendicitis in the pediatric age group [58]. In special populations such as preterm infants, abdominal conditions such as appendicitis present with non-specific symptoms; early diagnosis and treatment influence prognosis. Due to good echogenicity and absence of intra-abdominal fat in this age group, observation by USG is more straightforward in excluding conditions such as enterocolitis, Hirschsprung's disease, and meconium ileus [41].

On CT, an increased appendiceal diameter, irregular wall thickening, pericecal-retrocolic inflammation, and the arrowhead sign (thickening of the cecal wall around the root of the appendix) are some diagnostic features to look for. Using contrast allows for assessing appendiceal wall enhancement, differentiating pelvic vessels from the retrocecal appendix, and identifying other pathologies [99]. Contrast-enhanced CT (CECT) has a sensitivity and specificity of 88-100% and 92-98% respectively, in the diagnosis of SHA [31].

The subhepatic appendix can be elusive even to CECT, such that misdiagnosed patients have undergone cholecystectomy for gallstones and have reported back months later due to persistent symptoms. Only on performing a diagnostic laparoscopy or repeat CECT was conclusive evidence found [21,37]. Perhaps this sheds light on our overdependence on imaging to such an extent that it has lowered our degree of suspicion of aberrancies. Delays in appendectomy by over 24-36 hours can increase the complication rate, such as perforation and abscess, reiterating the importance of prompt diagnosis [103].

Surgical Approach

The use of laparoscopy for diagnosis and therapy has been recommended for managing SHA, where non-invasive imaging may be misleading. Laparoscopy, due to the flexible nature of port placements, allows for inspection of various appendiceal positions and the exclusion of other organ pathologies [4]. It improves diagnostic specificity in females of reproductive age who may present with gynecologic pathologies having overlapping symptoms [104]. Grassi et al. reported a case of subhepatic perforated appendix due to toothpick ingestion, which was infixed to the liver. The use of laparoscopy in the above case enabled the easy retrieval of the foreign body without causing hepatic parenchymal damage, reiterating its ability to provide operative fluidity in novel situations [105]. Laparoscopic appendectomy has found a role in traditionally openly treated cases, such as perforation and abscesses, with lesser postoperative complications, provided the center has sufficient expertise. This is of importance as most cases of SHA present with complications. The removal of infected material using an endobag reduces trocar site infections, and postoperative intra-abdominal abscesses can be prevented if the appendiceal stump is thoroughly evaluated, followed by extensive irrigation and drainage [106]. In accordance, laparoscopically treated cases of SHA reported early mobilization, better cosmetic appearance, and faster discharges.

In certain instances, when unable to mobilize the appendix laparoscopically due to dense adhesions, laparoscopic appendectomy was converted to an open procedure for direct access and better tactile input [55,76,79]. Muhamad Zin et al. were prompted to perform a right hemicolectomy with primary anastomosis when unable to mobilize the cecum adherent to the appendix and posterior parietal peritoneum [79]. Another approach used in the case of dense adhesions during laparoscopy was to place a subhepatic drain initially and then perform an interval appendectomy. Certain recommendations to prevent conversion to laparotomy in difficult situations include the use of angled telescopes, extra port placement, the twisting method for long appendices, hydrodissection, and early cecal mobilization [107]. The methodology and laparoscopic port placement should be customized to every patient, adhering to the principles of triangulation and ergonomics [4]. However, in complicated cases, conversion may be inevitable when direct access is necessary. This should not be viewed as a failure, as patient safety is prioritized first.

Limitations

Although this review synthesizes the best available evidence on SHA, important limitations should be acknowledged. Firstly, the evidence base is composed entirely of case reports and small case series, which are inherently subject to publication and selection biases that cannot be evaluated statistically. Unusual, complicated, or educational cases are more likely to be published than typical presentations. Secondly, the clinical, imaging, operative, and postoperative details were inconsistently documented, necessitating an available-case approach with variable denominators. Residual publication bias was inevitable as our literature search was restricted to PubMed, Scopus, and Google Scholar, and several eligible reports published after the search date were identified through citation chaining. Moreover, the cases span multiple diagnostic and operative eras. Some observed patterns, such as CT more frequently preceding laparoscopic appendectomy or shorter postoperative stays after laparoscopy, likely reflect temporal differences in diagnostic availability and perioperative protocols rather than intrinsic differences between case subsets. Follow-up in most reports was short, precluding assessment of long-term outcomes. Lastly, generalizability is limited because published cases predominantly originate from surgical centers, whereas non-operative, outpatient, or conservatively managed SHA cases are likely under-represented or absent from the literature. Nonetheless, within these constraints, the review consolidates the available clinical experience with SHA, highlights consistent diagnostic features and pitfalls, and identifies priorities for prospective studies and standardized case reporting.

## Conclusions

Effective diagnosis and management of SHA requires integrating clinical suspicion with judicious use of imaging and timely surgical intervention. We recommend that SHA be considered in patients presenting with right hypochondriac or atypical abdominal pain, particularly when hepatobiliary investigations are inconclusive. USG remains a reasonable first-line modality and assists initial evaluation, but has limited sensitivity for atypically located appendices. CT, especially CECT, should be pursued if ultrasound is non-diagnostic, as it offers superior preoperative detection. Laparoscopy provides definitive diagnostic clarification when imaging remains inconclusive and simultaneously serves as an avenue for therapeutic intervention. However, surgeons should remain prepared to convert to an open appendectomy when dense adhesions or anatomical anomalies are encountered. While published cases generally report favorable outcomes, the clinical course of SHA likely varies widely, thus reinforcing the importance of early recognition and tailored operative management. As far as future research is concerned, it should first focus on establishing structured diagnostic algorithms for atypical appendicitis, incorporating both imaging pathways and surgical decision-making to reduce diagnostic delays. In addition, multicenter registries or prospective studies can better define the true incidence, identify consistent diagnostic pitfalls, and characterize both short- and long-term outcomes of SHA. Finally, anatomical and embryological studies investigating cecal descent and other congenital anomalies associated with SHA can strengthen clinical suspicion and surgical planning.

## Appendices

Appendix A 

**Table 4:** Detailed results of the systematic review

Sex (n = 97)	Number (%) of patients
Male	67 (69.07)
Female	30 (30.93)
Age (n = 93)	Years
Range	0.0055 (2 days) - 71
Mean	30.56 (S.D = 18.02)
Median	29
Age Category (n = 96)	Number (%) of patients
*Adults*	68 (70.83)
Male	47 (48.96)
Female	21 (21.88)
* Elderly*	5 (5.21)
* Paediatric*	24 (29.17)
Male	19 (19.79)
Female	9 (9.38)
Infants	1 (1.04)
Neonates	1 (1.04)
Medical History/Comorbidities Reported (n = 58)	Number (%) of patients
None	33 (56.90)
History of Surgery	8 (13.79)
History of Abdominal Surgery	8 (13.79)
History of Cholecystectomy	5 (8.62)
History of: Cholangitis with Choledocholithiasis; perforated cholecystitis; Intestinal Malrotation; percutaneous cholecystotomy, biliary sphincterotomy and stent placement; Left breast cyst excision; Spleen conserving surgery; Left colectomy; Benign Prostatic Hyperplasia; Simple Ovarian Cyst	1 (1.72) Each
Hypertension	7 (12.07)
Diabetes Mellitus	7 (12.07)
Obesity	3 (5.17)
Chronic Dyspepsia	2 (3.44)
Chronic Obstructive Pulmonary Disease	2 (3.44)
History Of Peptic Ulcer	2 (3.44)
Asthma	2 (3.44)
History of: Ongoing pregnancy; Chronic Iron-Deficiency Anemia; Dyslipidaemia, Inflammatory Bowel Syndrome; Anxiety; Hypothyroidism; Fibromyalgia; Pyelonephritis; Ischemia Heart Disease; Alcoholism; Colon Cancer; Ischemic Heart Disease	1 (1.72) Each
Symptoms (n = 96)	Number (%) of patients
Pain	93 (96.88)
Site of Pain (n = 93)	Number (%) of patients
Abdomen	91 (97.85)
Right Flank	8 (8.60)
Back	5 (5.38)
Chest	2 (2.15)
Duration of Pain (n = 93)	Number (%) of patients
Acute	81 (87.10)
Chronic	5 (5.38)
Acute-on-Chronic	1 (1.08)
Not Reported	6 (2.08)
Site of Abdominal Pain (n = 82)	Number (%) of patients
Right Hypochondrium	67 (81.71)
Right Upper Quadrant	63 (76.83)
Right Iliac Fossa	44 (53.36)
Right Lower Quadrant	38 (46.34)
Right Lumbar region	32 (39.02)
Right Abdomen	30 (36.59)
Periumbilical region	29 (35.37)
Epigastrium	26 (31.71)
Diffuse/Generalised	12 (14.63)
Nature of Pain (n = 52)	Number (%) of patients
*Migratory*	16 (30.77)
Periumbilical/Umbilical to Right Upper Quadrant	10 (62.50)
Periumbilical/Umbilical to Right Lower Quadrant	9 (56.25)
Periumbilical to Right Flank	2 (12.50)
Upper Abdomen to Right Iliac Fossa	1 (7.69)
*Radiating*	10 (19.23)
To the back	3 (30.00)
To the Right Flank	3 (30.00)
To the Right Iliac Fossa	3 (30.00)
To the Lower abdomen; Right Shoulder; Lower chest; Right upper abdomen	1 (10.00) Each
Continuous/Persistent/Constant	9 (17.31)
Colicky	8 (15.38)
Recurrent	6 (11.54)
Progressive in Intensity	7 (13.46)
Dull	5 (9.62)
Intermittent	3 (5.77)
Cramping	3 (5.77)
Aching	3 (5.77)
Stabbing; Referred; Pleuritic; Dragging; Post-prandial	1 (1.92) Each
* Vomiting/Regurgitation*	66 (68.75)
Fever	45 (46.88)
Nausea	32 (33.33)
Loss of Appetite	19 (19.79)
Diarrhoea	6 (6.25)
Constipation	5 (5.21)
Chills	3 (3.13)
Abdominal Distension/Bloating	3 (3.13)
Dysuria	3 (3.13)
Obstipation	2 (2.08)
Breathlessness	2 (2.08)
Clinical Signs (n = 92)	Number (%) of patients
Tenderness (n = 84)	84 (91.30)
Right Hypochondrium	67 (79.76)
Right Upper Quadrant	50 (59.52)
Right Iliac Fossa	38 (45.24)
Right Lumbar Region	30 (35.71)
Right Lower Quadrant	26 (30.95)
Right Abdomen	22 (26.19)
Epigastrium	18 (21.43)
Umbilical/Periumbilical region	14 (16.67)
Generalised/Diffuse	10 (11.90)
Right Flank	5 (5.95)
Rebound Tenderness (n = 26)	26 (28.26)
Right Iliac Fossa	17 (65.38)
Right Hypochondrium	16 (61.54)
Right Upper Quadrant	14 (53.85)
Right Lower Quadrant	10 (38.46)
Right Lumbar	9 (34.62)
Right Abdomen	5 (19.23)
Right Flank	1 (3.85)
Guarding (n = 30)	30 (32.61)
Right Hypochondrium	21 (70.00)
Right Upper Quadrant	18 (60.00)
Right Iliac Fossa	19 (63.33)
Right Lower Quadrant	16 (53.33)
Right Lumbar	15 (50.00)
Right Abdomen	13 (43.33)
Epigastrium	11 (36.67)
Generalised	6 (20.00)
Right Flank	1 (3.33)
Febrile	37 (40.22)
Tachycardia	22 (23.91)
Hypotension	6 (6.52)
Tachypnoea	9 (9.78)
Psoas Sign	7 (7.61)
Dehydration	5 (5.43)
Decreased/absent bowel sounds	5 (5.43)
Murphy’s Sign	4 (4.35)
Abdominal Distension	4 (4.35)
Right Hypochondrial mass	4 (4.35)
Jaundice/Icterus	3 (3.26)
Basal Crackles/Crepitations	2 (2.17)
Right Renal Angle Tenderness/Murphy’s Punch Positive	1 (1.09)
Laboratory Investigations (n = 88)	
WBC Count (n = 68)	Cells/mm^3^
Range	3000 – 28100
Mean	14484 (S.D = 4775)
Reporting of Leucocytosis (n = 84)	Number (%) of patients
Present	64 (76.19)
Reporting of Neutrophilia (n = 87)	
Present	38 (43.68)
Reporting of CRP	Number (%) of patients
Reported	21 (23.86)
Range (mg/L)	12.4 – 347
Mean (mg/L)	147 (S.D = 117.2)
Hyperbilirubinemia	7 (7.95)
Conjugated Hyperbilirubinemia	3 (3.41)
Anemic	5 (5.68)
Elevated SGPT/ALT	3 (3.41)
Elevated Platelet Count	3 (3.41)
Elevation of both AST and ALT	2 (2.27)
Elevated Alkaline Phosphatase; Raised INR; Erythrocytosis; Hemoconcentration; Elevated Lipase; Hyperlactatemia; Metabolic Acidosis; Hematuria; Decreased serum ferritin, iron and iron binding capacity	1 (1.14) Each
X-Ray Reported (n = 33)	33 (34.02)
X-Ray Kidneys, Ureters, and Bladder	1 (3.03)
Free Air Under the Diaphragm	3 (9.09)
Chest X-Ray (n =19)	19 (57.58)
Abnormal	9 (47.37)
Patchy consolidation in right upper lung zone; Right lower pulmonary lobe infiltrate; Elevation of right hemidiaphragm; Right subdiaphragmatic abscess; Prominent Lung Markings; Preexisting right basal collapse (stable in size)	1 (5.26) Each
Abdominal X-Ray (n = 21)	21 (63.64)
Abnormal	11 (52.38)
Dilated Bowel Loops	6 (28.57)
Air/Fluid Levels in Bowel	3 (14.29)
Opacity in Right Hypochondrium/RUQ	3 (14.29)
Gas-filled bowel loops	3 (14.29)
Dilated Stomach	1 (4.76)
Ultrasound Abdomen (n = 78)	Number (%) of patients
Abnormal Study	61 (78.21)
Inflamed Appendix	27 (34.62)
Appendix located Subhepatically	21 (26.92)
Collection/Free Fluid in Right Subhepatic Space/Morison's Pouch	13 (16.67)
Possibility of Abscess	7 (8.97)
Suggestive of Cholecystitis	8 (10.81)
Inconclusive Study	6 (7.69)
Possibility of Liver Abscess	4 (5.13)
Appendix Dilated	21 (26.92)
Collection/Fluid in Right Iliac Fossa	7 (8.97)
Presence of Gallstones	5 (6.41)
Appendicolith	7 (8.97)
Contracted Gall Bladder	3 (3.85)
Prominent Lymph Nodes in the Right Lower Quadrant	3 (3.85)
Enlarged Mesenteric Lymph Nodes	3 (3.85)
Collection/Fluid in Pelvis	3 (3.85)
Perforation of Appendix	2 (2.56)
Hepatic Steatosis	3 (3.85)
Right perinephric fluid collection; Right Subdiaphragmatic Abscess; Hepatomegaly; Hepatic cysts; Distended stomach; Distended first and second parts of Duodenum; Distended Gall Bladder; Distended Terminal Ileum; Multiple right renal calculi; Ileus; Bowel Edema; Gall Bladder polyp; Small and contracted Gall Bladder; Right Paracolic collection; Prominent bilateral renal collecting system; minimal ascites in the left iliac fossa and pelvis; Bilateral pleural effusion	1 (1.28) Each
Computed Tomography (CT) (n = 52)	Number (%) of patients
Type of Study (n = 52)	
Contrast-Enhanced	33 (71.15)
Plain	10 (19.23)
Not Reported	5 (9.62)
Subhepatic Appendix Reported	42 (80.77)
Appendix Thickened/Dilated	22 (42.31)
Subhepatic/Undescended Caecum	15 (28.85)
Periappendiceal Fat Stranding	19 (36.54)
Appendicolith	15 (28.85)
Appendix Perforated	11 (21.15)
Collection in Right Subhepatic Space	10 (19.23)
Retrocecal Appendix	11 (21.15)
Presence/Suspicion of Malrotation	4 (7.69)
*Presence of Abscess*	7 (13.46)
Liver	3 (42.86)
Subhepatic	2 (28.57)
Perihepatic; Right Paracolic Gutter; Right Empyema Thoracis; Right Subdiaphragmatic; Pelvic	1 (14.29) Each
Pelvic Collection	5 (9.62)
Gangrenous Appendix; Peritoneal effusion; Absence of Right Kidney; Left Hydronephrosis; Hypertrophied Left Kidney; Bilateral Kidney Stones; Thickening of Gerota's Fascia; Hepatomegaly; Intestinal Obstruction; Colitis; Right Basal Consolidation (Lung)	1 (1.92) Each
Magnetic Resonance Imaging (MRI) (n = 2)	
Gall Bladder Not Visualised (MRCP)	1
Paracolic abscess	1
Right Ovarian Cyst	1
Preoperative Diagnoses (n = 89)	Number (%) of patients
Appendicitis	69 (77.53)
Subhepatic Appendicitis	58 (65.17)
Cholecystitis	25 (28.09)
Liver Abscess	7 (7.87)
Peritonitis For Evaluation	4 (4.76)
Pyelonephritis	3 (3.37)
Gastroenteritis	3 (3.57)
Acute Abdomen	3 (3.57)
Renal Colic	3 (3.57)
Perforated Peptic Ulcer	2 (2.25)
Urinary Tract Infection	2 (2.25)
Paracolic Abscess	2 (2.25)
Pneumonia	2 (2.25)
Gall Bladder Perforation	2 (2.25)
Subdiaphragmatic Abscess	2 (2.25)
Paracolic Abscess	2 (2.25)
Hollow Viscus Perforation	2 (2.25)
Perforated Gastric Ulcer; Duodenal Perforation; Acute gastritis; acute epiploic appendagitis; Hepatitis; Pancreatitis; Right Renal Calculi; Psoas Abscess; Perinephric abscess; Ileus of Transverse Colon; Acute angulation; Torsion of Ovarian Cyst; Biliary Colic; Sepsis; Right Empyema Thoracis; Gall Bladder Polyp; Cholelithiasis	1 (1.12) Each
Confirmatory Diagnostic Method (n = 97)	
* *Computed Tomography (CT)	39 (40.21)
Confirmatory CT Leading to Successful Laparoscopic Appendectomy (n = 39)	
Yes	20 (51.28)
No	14 (35.90)
Management/Specific Surgery-Type Not Reported	5 (12.82)
*Confirmatory CT Leading to Open Appendectomy (n = 39)*	Number (%) of patients
Yes	12 (30.76)
No	22 (56.41)
Management/Specific Surgery-Type Not Reported	5 (12.82)
*Contrast-Enhanced CT (CECT)*	29 (74.36), (29.90)
*Confirmatory CECT Leading to Successful Laparoscopic Appendectomy (n = 29)*	
Yes	14 (48.28)
No	12 (41.38)
Management/Specific Surgery-Type Not Reported	3 (10.34)
Confirmatory CECT Leading to Open Appendectomy (n = 29)	
Yes	10 (34.48)
No	16 (55.17)
Management/Specific Surgery-Type Not Reported	3 (10.34)
Plain CT	8 (20.51), (8.25)
* Confirmatory Plain CT Leading to Successful Laparoscopic Appendectomy (n = 8)*	
Yes	4 (40.00)
No	4 (40.00)
Management/Specific Surgery-Type Not Reported	2 (20.00)
Confirmatory Plain CT Leading to Open Appendectomy (n = 8)	
Yes	4 (50.00)
No	2 (25.00)
Management/Specific Surgery-Type Not Reported	2 (25.00)
CT- Type Not Reported	2 (5.13), (2.06)
Ultrasound Abdomen	18 (18.56)
*Confirmatory USG leading to Successful Laparoscopic Appendectomy (n = 18)*	
Yes	7 (38.89)
No	8 (44.44)
Management/Specific Surgery-Type Not Reported	3 (16.67)
*Confirmatory USG leading to Open Appendectomy (n = 18)*	
Yes	8 (44.44)
No	7 (38.89)
Management/Specific Surgery-Type Not Reported	3 (16.67)
X-Ray Abdomen	1 (1.03)
Intraoperatively	38 (39.18)
Laparoscopically	13 (34.21), (13.40)
Via Laparotomy	25 (65.79), (25.77)
Autopsy	1 (1.03)
Management (n = 97)	Number (%) of patients
Medical/Conservative	3 (3.09)
Surgical	93 (95.88)
Not Reported	1 (1.03)
Type of Appendectomy Reported (n = 85)	(91.40)
Laparoscopic Appendectomy Attempted	44 (51.76)
Laparoscopic Appendectomy Converted to Open Appendectomy	7 (15.91)
Open Appendectomy	48 (56.47)
* Incisional Approach (n = 48)*	
Not Reported	20 (41.67)
Midline	12 (25.00)
Gridiron/McBurney’s	5 (10.42)
Right Paramedian (All Right Upper Rectus)	3 (6.25)
Rocky Davis; Rutherford Morrison’s; Transverse Right Lumbar; Transverse Right Lower Quadrant; Transverse Subumbilical; Incision at the level of the umbilicus; Transverse Right Upper Flank; Right Pararectal	1 (2.08) Each
Extension of Incision	5 cases
Interval Appendectomy	3 (3.52)
Cholecystectomy	3 (3.52)
Right Hemicolectomy	1 (1.18)
Surgical Removal of Meckel’s Diverticulum	2 (2.35)
Partial Omentectomy; Right Sided Thoracotomy; Ileocecal resection with side-to-side ileoascendostomy	1 (1.18) Each
*Number of Ports Used in Successful Laparoscopic Appendectomy (n = 37)*	Number (%) of patients
Not Reported	24 (64.86)
3	8 (21.62)
4	3 (8.11)
2	1 (2.70)
Peritoneal Toileting/Lavage	23 (27.06)
*Placement of Surgical Drain*	24 (28.24)
Pelvis	7 (29.17)
Subhepatic	6 (25.00)
Right Upper Quadrant	2 (8.33)
Pouch of Douglas	2 (8.33)
Pericecal; Suprapubic; Right Pelvis; Left Pelvis	1 (4.17)
Not Reported	10 (41.67)
Intraoperative Findings (n = 85)	Number (%) of patients
Presence of Adhesions	33 (38.82)
Perforated Appendix	26 (30.59)
Presence of Abscess/Pus (n = 26)	26 (30.59)
Subhepatic Space/Morison’s Pouch	14 (53.85)
Right Subdiaphragmatic	4 (15.38)
Pelvis	3 (11.54)
Pouch Of Douglas	2 (7.69)
Generalised Over the Abdomen	2 (7.69)
Unspecified	2 (7.69)
Liver; Right hypochondrium; Right Upper Quadrant; Right Paracolic Gutter; Subcapsule of Liver; Right Empyema Thoracis; Perirenal	1 (3.85) Each
Retrocecal Appendix	20 (23.53)
Gangrenous Appendix	12 (14.12)
Presence of Appendicolith	10 (11.76)
Suppurative Appendix	2 (2.35)
Meckel’s Diverticulum	2 (2.35)
Dilated Transverse Colon	2 (2.35)
Absence of Ascending Colon; Sloughed off appendix; Partly necrosed appendix; Left dolichocolon; Incomplete common mesentery; Sigmoid Colon in Right Iliac Fossa	1 (1.18) Each
Short Ascending Colon	7 (8.24)
Peritonitis	6 (7.06)
Presence of Pyogenic Membrane	2 (2.35)
Bacterial Culture of Pus (n = 8)	
* Organism Isolated*	
Escherichia coli	All 8 cases
E. coli alone	6 (75.00)
E. coli, Klebsiella pneumoniae, Proteus Mirabilis, and Enterococcus; E. coli, Klebsiella oxytoca, Peptostreptococcus anaerobius, and Prevotella melaninogenica	1 (12.50) Each
Antibiotic Sensitivity (n = 6)	
Amoxicillin-Clavulanic Acid	4 (66.67)
Metronidazole	3 (50.00)
Gentamicin	2 (33.33)
Postoperative Complications (n = 83)	Number (%) of patients
None	72 (86.75)
Surgical Site Infection	3 (3.61)
Ileus; Chronic abdominal pain; Constipation; Intestinal obstruction; Urinary Tract Infection; Pyogenic Liver and Subhepatic abscess; Septic Shock; Hernia; Spinal Headache; Ruptured retroperitoneal abscess	1 (1.20) Each
Histopathology Report (n = 41)	Number (%) of patients
Acute Appendicitis	28 (68.29)
Suppurative Appendicitis	7 (17.07)
Perforated Appendix	4 (9.76)
Periappendicitis	4 (9.76)
Mucosal Ulceration/Erosion	4 (9.76)
Chronic Appendicitis	2 (4.88)
Acute-on-Chronic Appendicitis	2 (4.88)
Gangrenous Appendicitis	2 (4.88)
Pan-parietal/transmural inflammation	2 (4.88)
Necrotic Appendix; Appendiceal diverticulum; Diffuse large B-cell lymphoma; Heterotopic gastric mucosa in Meckel's Diverticulum; Well-differentiated appendiceal neuroendocrine neoplasm; Epiploic appendagitis	1 (2.44) Each
Outcomes (n = 85)	Number (%) of patients
Discharged	84 (98.82)
Died	1 (1.18)
Postoperative Day of Discharge (n = 55)	
Range	1^st^ – 32^nd^ day
Mean	5.13 days (S.D = 5.19)
Mode	2^nd^ day (n =11)
Median	4^th^ day
Interquartile Range	4 days
Postoperative Day of Discharge for Laparoscopic Appendectomy (n = 20)	
Range	1^st^ – 9^th^ day
Mean	3.1 days (S.D = 2.25)
Mode	2^nd^ day (n = 6)
Median	2^nd^ day
Interquartile Range	2.5 days
Postoperative Day of Discharge for Open Appendectomy (n = 35)	
Range	1^st^ – 32^nd^ day
Mean	6.11 days (S.D = 6.08)
Mode	5^th^ day (n = 7)
Median	5^th^ day
Interquartile Range	4.5 days

Appendix B 

**Table 5:** Detailed results of cases of subhepatic appendicitis with documented congenital anatomical anomalies

Sex (n = 51)	Number (%) of patients
Male	38 (74.51)
Female	13 (25.49)
Age (n = 50)	Years
Range	7-71
Mean	33.28 (SD = 16.48)
Median	31.5
Age Category (n = 51)	Number (%) of patients
*Adults*	42 (82.35)
Male	30 (71.43)
Female	12 (28.57)
*Elderly*	3 (5.88)
*Paediatric*	9 (17.65)
Male	8 (88.89)
Female	1 (11.11)
Medical History/Comorbidities Reported (n = 28)	
None	17 (60.71)
History of Surgery	4 (14.29)
History of Abdominal Surgery	4 (14.29)
History of Cholecystectomy	3 (10.71)
History of: Left Colectomy; Cholangitis with Choledocholithiasis; perforated cholecystitis; Intestinal Malrotation; percutaneous cholecystotomy, biliary sphincterotomy and stent placement, Benign Prostatic Hyperplasia; Simple Ovarian Cyst; Intestinal Malrotation	1 (3.57) Each
Chronic Dyspepsia	2 (7.14)
Diabetes Mellitus	2 (7.14)
Hypertension	2 (7.14)
Obesity	2 (7.14)
History of: Chronic Iron Deficiency Anemia; Chronic Obstructive Pulmonary Disease; Alcoholism; Ongoing pregnancy; Colon Cancer; Anxiety; Hypothyroidism; Fibromyalgia; Ischemic Heart Disease	1 (3.57) Each
Symptoms (n = 51)	Number (%) of patients
Pain	50 (98.04)
Site of Pain (n = 50)	Number (%) of patients
Abdomen	50 (100.00)
Right Flank	5 (10.00)
Back	1 (2.00)
Site of Abdominal Pain (n = 46)	Number (%) of patients
Right Hypochondrium	35 (76.09)
Right Upper Quadrant	32 (69.57)
Right Iliac Fossa	23 (50.00)
Right Lumbar	17 (36.96)
Right Lower Quadrant	20 (43.48)
Epigastrium	15 (32.61)
Right Abdomen	15 (32.61)
Periumbilical region	14 (30.43)
Diffuse/Generalised	5 (10.87)
Nature of Pain (n = 28)	Number (%) of patients
*Migratory*	9 (32.14)
Periumbilical/Umbilical to Right Upper Quadrant	6 (66.67)
Periumbilical/Umbilical to Right Lower Quadrant	5 (55.56)
Periumbilical to Right Flank	1 (11.11)
*Radiating*	5 (17.86)
To the Right Flank	2 (40.00)
To the Right Iliac Fossa	2 (40.00)
To the Lower Abdomen; Back	1 (10.00) Each
Continuous/Persistent/Constant	6 (21.43)
Recurrent	3 (10.71)
Progressive in Intensity	4 (14.29)
Dull	3 (10.71)
Colicky	2 (7.14)
Intermittent	2 (7.14)
Aching	2 (7.14)
Cramping; Post-prandial; Dragging	1 (3.57) Each
Vomiting/Regurgitation	33 (64.71)
Fever	19 (37.25)
Nausea	21 (41.18)
Loss of Appetite	12 (23.53)
Diarrhoea	2 (3.92)
Constipation	3 (5.88)
Chills	2 (3.92)
Obstipation	2 (3.92)
Abdominal Distension/Bloating; Dysuria	1 (1.96) Each
Clinical Signs (n = 49)	Number (%) of patients
Tenderness (n = 46)	46 (93.88)
Right Hypochondrium	40 (86.96)
Right Upper Quadrant	25 (54.35)
Right Iliac Fossa	17 (36.96)
Right Lumbar Region	17 (36.96)
Right Lower Quadrant	13 (28.26)
Right Abdomen	12 (26.09)
Epigastrium	12 (26.09)
Umbilical/Periumbilical region	7 (15.22)
Generalised/Diffuse	7 (15.22)
Right Flank	2 (4.35)
Rebound Tenderness (n = 16)	16 (32.65)
Right Iliac Fossa	9 (56.25)
Right Hypochondrium	10 (62.50)
Right Upper Quadrant	8 (50.00)
Right Lower Quadrant	5 (31.25)
Right Lumbar	5 (31.25)
Right Abdomen	3 (18.75)
Right Flank	1 (6.25)
Guarding (n = 16)	16 (32.65)
Right Hypochondrium	11 (68.75)
Right Upper Quadrant	9 (56.25)
Right Iliac Fossa	8 (53.33)
Right Lower Quadrant	9 (56.25)
Right Lumbar	7 (43.75)
Right Abdomen	6 (37.50)
Epigastrium	5 (31.25)
Generalised; Right Flank	1 (6.25) Each
Febrile	17 (34.69)
Tachycardia	14 (28.57)
Hypotension	4 (8.16)
Tachypnoea	3 (6.12)
Psoas Sign	2 (4.08)
Dehydration	5 (10.20)
Decreased/absent bowel sounds	4 (8.16)
Murphy’s Sign	2 (4.08)
Abdominal Distension	4 (8.16)
Right Hypochondrial mass	3 (6.12)
Jaundice/Icterus	2 (4.08)
Basal Crackles/Crepitations	1 (2.04)
Laboratory Investigations (n = 45)	
WBC Count (n = 34)	Cells/mm^3^
Range	3000 – 25500
Mean	14308 (S.D = 4654)
Reporting of Leucocytosis (n = 45)	Number (%) of patients
Present	36 (80.00)
Reporting of Neutrophilia (n = 45)	
Present	19 (42.22)
Reporting of CRP	Number (%) of patients
Reported	7 (15.56)
Range (mg/L)	28 – 347
Mean (mg/L)	162 (S.D = 119.1)
Hyperbilirubinemia	3 (6.67)
Conjugated Hyperbilirubinemia	1 (2.22)
Anemia; Elevated SGPT/ALT; Elevated Alkaline Phosphatase; Metabolic acidosis; Decreased serum ferritin, iron and iron binding capacity	1 (2.22)
X-Ray Reported (n = 19)	19 (37.25)
X-Ray Kidneys, Ureters and Bladder	1 (5.26)
Free Air Under the Diaphragm	2 (10.53)
Chest X-Ray (n = 10)	10 (52.63)
Abnormal	3 (30.00)
Prominent Lung Markings; Preexisting Right Basal Collapse (stable in size)	1 (5.26) Each
Abdominal X-Ray (n = 12)	12 (63.16)
Abnormal	4 (33.33)
Dilated Bowel Loops; Opacity in Right Hypochondrium	1 (28.57)
Ultrasound Abdomen (n = 38)	Number (%) of patients
Abnormal Study	28 (73.68)
Inflamed Appendix	9 (23.68)
Appendix located Subhepatically	7 (18.42)
Collection/Free Fluid in Right Subhepatic Space/Morison's Pouch	7 (18.42)
Possibility of Abscess	4 (10.53)
Suggestive of Cholecystitis	4 (10.53)
Inconclusive Study	2 (5.26)
Possibility of Liver Abscess	3 (7.89)
Appendix Dilated	5 (13.16)
Collection/Fluid in Right Iliac Fossa	4 (10.53)
Contracted Gall Bladder	3 (7.89)
Presence of Gallstones	2 (5.26)
Appendicolith	2 (5.26)
Collection/Fluid in Pelvis	2 (5.26)
Perforation of Appendix	1 (2.63)
Prominent Lymph Nodes in Right Lower Quadrant; Enlarged Mesenteric Lymph Nodes; Hepatic cysts; Hepatomegaly; Hepatic Steatosis; Distended stomach; Distended duodenum; Small and contracted Gall bladder; Ileus	1 (2.63) Each
Computed Tomography (CT) (n = 30)	Number (%) of patients
Type of Study (n = 30)	
Contrast-Enhanced	24 (80.00)
Plain	3 (10.00)
Not Reported	3 (10.00)
Subhepatic Appendix Reported	25 (83.33)
Appendix Thickened/Dilated	11 (36.67)
Subhepatic/Undescended Caecum	15 (50.00)
Periappendiceal Fat Stranding	10 (33.33)
Appendicolith	7 (23.33)
Appendix Perforated	8 (26.67)
Collection in Right Subhepatic Space	7 (23.33)
Retrocecal Appendix	6 (20.00)
Presence/Suspicion of Malrotation	4 (13.33)
Presence of Abscess	3 (10.00)
Liver	2 (66.67)
Subhepatic	2 (66.67)
Perihepatic; Right Paracolic Gutter	1 (33.33) Each
Pelvic Collection	3 (10.00)
Absence of Right Kidney; Left Hydronephrosis; Hypertrophied Left Kidney	1 (3.33) Each
Magnetic Resonance Imaging (MRI) (n = 1)	
Gall Bladder Not Visualised (MRCP)	1
Preoperative Diagnoses (n = 45)	Number (%) of patients
Appendicitis	36 (80.00)
Subhepatic Appendicitis	31 (68.89)
Cholecystitis	17 (37.78)
Liver Abscess	3 (6.67)
Duodenal Perforation	2 (4.44)
Peritonitis	2 (4.44)
Hollow Viscus Perforation; Gall bladder perforation; Acute Abdomen; Gastroenteritis; Perforated Peptic Ulcer; Renal Colic; Acute Angulation; Perinephric abscess; Paracolic abscess	1 (2.22) Each
Confirmatory Diagnostic Method (n = 51)	
Computed Tomography (CT)	25 (49.02)
*Confirmatory CT Leading to Successful Laparoscopic Appendectomy (n = 25)*	Number (%) of patients
Yes	15 (60.00)
No	9 (36.00)
Management/Specific Surgery-Type Not Reported	1 (4.00)
Confirmatory CT Leading to Open Appendectomy (n = 25)	Number (%) of patients
Yes	9 (36.00)
No	15 (60.00)
Management/Specific Surgery-Type Not Reported	1 (4.00)
Contrast-Enhanced CT (CECT)	21 (84.00), (41.18)
Confirmatory CECT Leading to Successful Laparoscopic Appendectomy (n = 21)	
Yes	12 (57.14)
No	8 (38.10)
Management/Specific Surgery-Type Not Reported	1 (4.76)
*Confirmatory CECT Leading to Open Appendectomy (n = 21)*	
Yes	8 (38.10)
No	12 (57.14)
Management/Specific Surgery-Type Not Reported	1 (4.76)
Plain CT	3 (12.00), (5.88)
Confirmatory Plain CT Leading to Successful Laparoscopic Appendectomy (n = 3)	
Yes	2 (66.67)
No	1 (33.33)
Confirmatory Plain CT Leading to Open Appendectomy (n = 3)	
Yes	1 (33.33)
No	2 (66.67)
CT- Type Not Reported	1 (4.00), (1.96)
Ultrasound Abdomen	6 (11.76)
*Confirmatory USG leading to Successful Laparoscopic Appendectomy (n = 6)*	
Yes	4 (66.67)
No	2 (33.33)
*Confirmatory USG leading to Open Appendectomy (n = 6)*	
Yes	2 (33.33)
No	4 (66.67)
Intraoperatively	19 (37.25)
Laparoscopically	6 (31.58), (11.76)
Via Laparotomy	13 (68.42), (25.49)
Autopsy	1 (1.96)
Management (n = 51)	Number (%) of patients
Medical/Conservative	1 (1.96)
Surgical	49 (96.08)
Not Reported	1 (1.96)
Type of Appendectomy Reported (n = 49)	Number (%) of patients
Laparoscopic Appendectomy Attempted	27 (55.10)
Laparoscopic Appendectomy Converted to Open Appendectomy	3 (11.11)
Open Appendectomy	25 (51.02)
Incisional Approach (n = 25)	Number (%) of patients
Not Reported	8 (32.00)
Midline	7 (28.00)
Gridiron/McBurney’s	4 (16.00)
Right Paramedian (All Right Upper Rectus)	3 (12.00)
Transverse Right Upper Flank; Right Pararectal; Transverse Right Lower Quadrant	1 (4.00) Each
Extension of Incision	4 cases
Interval Appendectomy	2 (4.08)
Cholecystectomy	2 (4.08)
Ileocecal resection with side-to-side ileoascendostomy	1 (2.04)
Number of Ports Used in Successful Laparoscopic Appendectomy (n = 24)	Number (%) of patients
Not Reported	15 (62.50)
3	2 (8.33)
4	6 (25.00)
2	1 (4.17)
Peritoneal Toileting/Lavage	14 (28.57)
Placement of Surgical Drain	14 (28.57)
Pelvis	2 (14.29)
Subhepatic	6 (42.88)
Left Pelvis; Pericecal; Right Upper Quadrant; Suprapubic	1 (7.69) Each
Not Reported	6 (42.88)
Intraoperative Findings (n = 47)	Number (%) of patients
Presence of Adhesions	20 (42.55)
Perforated Appendix	15 (31.91)
Presence of Abscess/Pus (n = 13)	13 (27.66)
Subhepatic Space/Morison’s Pouch	8 (61.54)
Pelvis	2 (15.38)
Pouch of Douglas	2 (15.38)
Right Subdiaphragmatic	2 (15.38)
Subcapsule of Liver; Right Upper Quadrant; Right Hypochondrium; Unspecified	1 (8.33) Each
Retrocecal Appendix	11 (23.40)
Gangrenous Appendix	6 (12.77)
Presence of Appendicolith	4 (8.51)
Dilated Transverse Colon	2 (4.26)
Suppurative Appendix; Partly necrosed appendix; Absence of ascending colon	1 (2.13) Each
Short Ascending Colon	7 (14.89)
Peritonitis	4 (8.51)
Presence of Pyogenic Membrane	2 (4.26)
Bacterial Culture of Pus (n = 1)	
Organism Isolated: Escherichia coli	1 case
Postoperative Complications (n = 49)	Number (%) of patients
None	40 (81.63)
Surgical Site Infection	2 (4.08)
Chronic abdominal pain; Constipation; Intestinal Obstruction; Urinary Tract Infection; Hernia; Spinal Headache	1 (2.04) Each
Histopathology Report (n = 23)	Number (%) of patients
Acute Appendicitis	17 (73.91)
Suppurative Appendicitis	3 (13.04)
Perforated Appendix	3 (13.04)
Mucosal Ulceration/Erosion	2 (8.70)
Chronic Appendicitis	2 (8.70)
Periappendicitis; Gangrenous appendicitis; acute-on-chronic appendicitis; Lymphoid hyperplasia; Pan-parietal/transmural inflammation	1 (4.35) Each
Outcomes (n = 48)	Number (%) of patients
Discharged	47 (97.92)
Died	1 (2.08)
Postoperative Day of Discharge (n = 31)	
Range	1^st^-20^th^ day
Mean	4.87 days (SD = 4.19)
Mode	2^nd^ day (n = 7)
Median	4^th^ day
Interquartile Range	3.5 days
Postoperative Day of Discharge for Laparoscopic Appendectomy (n = 20)	
Range	1^st^-7^th^ day
Mean	3.14 days (SD = 1.95)
Mode	2^nd^ day (n = 4)
Median	2.5 day
Interquartile Range	2.75 days
Postoperative Day of Discharge for Open Appendectomy (n = 35)	
Range	1^st^-20^th^ day
Mean	5.94 days (SD = 5.17)
Mode	5^th^ day (n = 5)
Median	5^th^ day
Interquartile Range	6 days
